# The micro-743a-3p–GSTM1 pathway is an endogenous protective mechanism against alcohol-related liver disease in mice

**DOI:** 10.1186/s11658-024-00557-x

**Published:** 2024-03-12

**Authors:** Tiantian Xu, Yan Pan, Qinchao Ding, Feiwei Cao, Kaixin Chang, Jiannan Qiu, Hui Zhuge, Liuyi Hao, Haibin Wei, Caijuan Si, Xiaobing Dou, Songtao Li

**Affiliations:** 1 School of Public Health, Zhejiang Chinese Medical University, Hangzhou, Zhejiang People’s Republic of China; 2 School of Life Science, Zhejiang Chinese Medical University, Hangzhou, Zhejiang People’s Republic of China; 3 Department of Clinical Nutrition, School of Medicine, Affiliated Zhejiang Hospital, Zhejiang University, Hangzhou, Zhejiang People’s Republic of China

**Keywords:** GSTM1, Alcohol-related liver disease, Hepatic steatosis, miR-743a-3p, ASK1

## Abstract

**Background and aims:**

Epidemiological evidence suggests that the phenotype of glutathione *S*-transferase mu 1 (GSTM1), a hepatic high-expressed phase II detoxification enzyme, is closely associated with the incidence of alcohol-related liver disease (ALD). However, whether and how hepatic GSTM1 determines the development of ALD is largely unclear. This study was designed to elucidate the role and potential mechanism(s) of hepatic GSTM1 in the pathological process of ALD.

**Methods:**

GSTM1 was detected in the liver of various ALD mice models and cultured hepatocytes. Liver-specific GSTM1 or/and micro (miR)-743a-3p deficiency mice were generated by adenoassociated virus-8 delivered shRNA, respectively. The potential signal pathways involving in alcohol-regulated GSTM1 and GSTM1-associated ALD were explored via both genetic manipulation and pharmacological approaches.

**Results:**

GSTM1 was significantly upregulated in both chronic alcohol-induced mice liver and ethanol-exposed murine primary hepatocytes. Alcohol-reduced miR-743a-3p directly contributed to the upregulation of GSTM1, since liver specific silencing miR-743a-3p enhanced GSTM1 and miR-743a-3p loss protected alcohol-induced liver dysfunctions, which was significantly blocked by GSTM1 knockdown. GSTM1 loss robustly aggravated alcohol-induced hepatic steatosis, oxidative stress, inflammation, and early fibrotic-like changes, which was associated with the activation of apoptosis signal-regulating kinase 1 (ASK1), c-Jun N-terminal kinase (JNK), and p38. GSTM1 antagonized ASK1 phosphorylation and its downstream JNK/p38 signaling pathway upon chronic alcohol consumption via binding with ASK1. ASK1 blockage significantly rescued hepatic GSTM1 loss-enhanced disorders in alcohol-fed mice liver.

**Conclusions:**

Chronic alcohol consumption-induced upregulation of GSTM1 in the liver provides a feedback protection against hepatic steatosis and liver injury by counteracting ASK1 activation. Down-regulation of miR-743a-3p improves alcohol intake-induced hepatic steatosis and liver injury via direct targeting on GSTM1. The miR-743a-3p–GSTM1 axis functions as an innate protective pathway to defend the early stage of ALD.

**Graphical Abstract:**

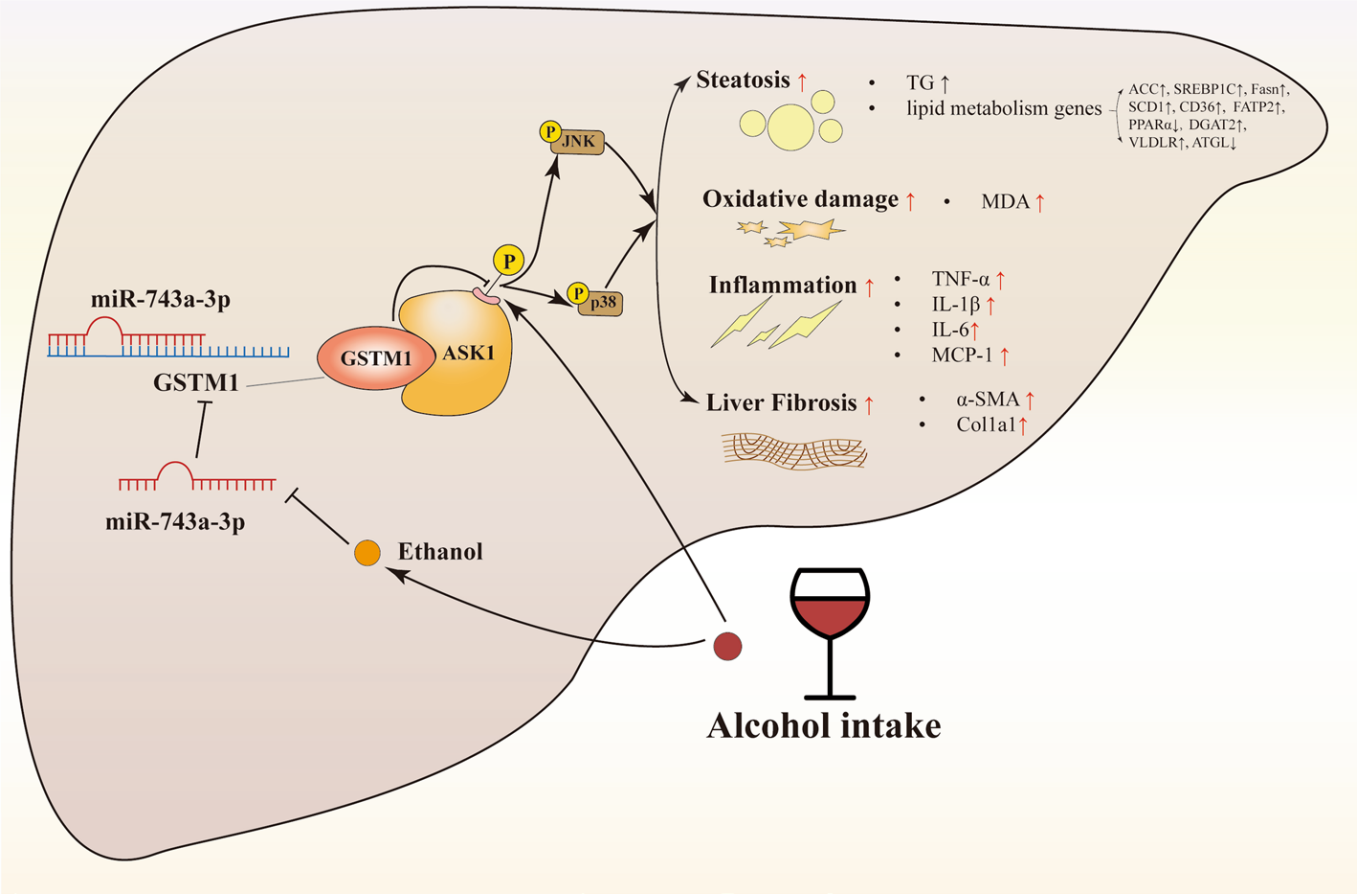

**Supplementary Information:**

The online version contains supplementary material available at 10.1186/s11658-024-00557-x.

## Introduction

Alcohol-related liver disease (ALD) has become a major cause of hepatogenic death worldwide, accounting for 47.9% of all liver cirrhosis deaths and 30% of all hepatocellular carcinoma (HCC) deaths [1]. ALD comprises a broad spectrum of pathologic stages ranging from simple hepatic steatosis, steatohepatitis, with some patients ultimately developing into liver fibrosis, cirrhosis, and even HCC [2, 3]. Although many progressions have been made to clarify the underlying mechanisms in ALD, there is still lack of clinically therapeutic way to cure the disease.

As a foodborne xenobiotic, alcohol cannot be stored in human body, meaning that all alcohol entering the human body must be completely metabolized and cleared by the body. The removal of xenobiotics is mainly completed by xenobiotic metabolizing enzymes, including phase I and phase II enzymes. For alcohol, the phase I enzymes, primarily alcohol dehydrogenase and cytochrome P450 2E1, are responsible for catalyzing ethanol to acetaldehyde, which is further metabolized to acetic acid by acetaldehyde dehydrogenase [4]. Although most phase II enzymes do not participate in alcohol catabolism, they may help the elimination of alcohol-induced harmful metabolites, such as lipid peroxidation products [5]. Moreover, it has been reported that the expression of several phase II enzymes, such as peroxiredoxin and heme oxygenase 1, etc., , were significantly disturbed in ALD, which contributes to the progression of ALD [6, 7]. However, it remains elusive as to how altered expression and/or activity of xenobiotic metabolizing enzymes in response to chronic alcohol consumption contributes to the pathogenesis of ALD.

Glutathione *S*-transferases (GSTs) are group of phase II xenobiotic metabolizing enzymes and widely distributed in different organisms, from single cell bacterium to plant, animal, and human being [8]. GSTs play an important role in the removal of toxic substances and antioxidant effects [9]. Eight classes of GSTs, including alpha (GSTA), kappa (GSTK), mu (GSTM), omega (GSTO), pi (GSTP), sigma (GST), theta (GSTT), and zeta (GSTZ), have been identified in mammals based on amino acid sequence and substrate specificity [10]. GSTMs are mainly responsible for facilitating glutathione conjugation reactions that eliminate endogenous and exogenous toxic compounds, especially electrophiles [11]. Currently, five subtypes of GSTM (1–5) have been identified in the liver, with GSTM1 being the highest expression in human liver [12, 13]. Epidemiological evidence showed that absolute gene absent polymorphism of GSTM1 could be detected in 33–63% East Asians, 38–67% of Caucasian, and 22–35% of Africans and of African Americans individuals [14–16]. GSTM1 null genotype was associated with an increased incidence of drug-induced liver injury [17–19]. People with GSTM1 null polymorphism exhibited a higher risk of ALD morbidity [20, 21], implying the importance of GSTM1 in ALD development. However, limited studies have been conducted to investigate the role of hepatic GSTM1 in ALD development.

In this study, our data uncovered that chronic alcohol consumption leads to a significant upregulation of hepatic GSTM1 expression, which is mediated by alcohol-induced downregulation of micro (miR)-743a-3p. Furthermore, we demonstrated that the genetic silencing of liver GSTM1 aggravates alcohol-induced hepatic steatosis via activating apoptotic signal-regulating kinase 1 (ASK1), c-Jun N-terminal kinase (JNK), and p38 signaling pathway. These data collectively suggest that alcohol-induced GSTM1 upregulation confers protection against ALD development.

## Materials and methods

### Animals

Animal procedures were approved by the Institutional Animal Care and Use Committee of Zhejiang Chinese Medical University (approval number 20220221-23). All mice were placed at 23 ± 2℃ and 55% ± 5% relative humidity for a 12 h light–dark cycle. Male C57BL/6J mice were fed a Lieber-DeCarli alcoholic liquid diet, or Lieber-DeCarli plus binge, as previously described [22, 23]. Single- and double-knockdown mice for liver specific GSTM1 and/or miR-743a-3p were generated by lateral tail vein injection with recombinant adenoassociated viral (AAV) serotype 8 gene transfer vectors bearing a hepatocyte-specific promoter (TBG) combination with mouse GSTM1 shRNA sequence (AAV8-GSTM1 KD) or/and miR-743a-3p whole length sequence (AAV8-miR-743a-3p KD). The detailed protocols are shown in Additional file 1.

### Hepatocytes

Isolation of mouse primary hepatocytes, construction of VL-17A, and culture of AML-12 were described previously [24–26]. Protocols for interventions, gene knockdown or overexpression, and miRNA mimics or inhibitor transfection were detailed in Additional file 1.

### Sample detections

Liver tissue was pretreated with NP-40 lysate, the samples were dissolved in assay buffer and vortexed extensively for 2 min as described previously [27], and then, TG content was determined according to the manufacturer’s instruction (Abcam, Cambridge, UK). The luciferase activity assay was carried out using the Dual-Luciferase Reporter Assay System (GenePharma, Shanghai, China). *HEK293T* cells were cotransfected with miR-743a-3p mimic/inhibitor and GSTM1-3′-UTR reporter. Luciferase activity was evaluated 48 h after transfection, as described previously [28]. The coimmunoprecipitation (co-IP) assay was performed as Sun, et al. described [29]. In brief, cells were lysed in co-IP buffer on ice for 30 min. Then, the cells were centrifuged, and the supernatant was collected, followed by incubation with Flag Magnetic Beads (Sigma-Aldrich, St. Louis, MO) with gentle rocking overnight at 4 °C. The mixture pelleted was washed three times and then eluted in a loaded buffer and denatured at 95 °C for 10 min before western blotting. The reagents and methods for malonaldehyde (MDA), GST activity, mRNA and protein expression, immunohistochemistry, and immunofluorescence are detailed in Additional file 1.

### Statistical analysis

GraphPad Prism (GraphPad Software 8.0.1) was used for statistical analysis. Data are presented as mean ± standard deviation (SD). One-way analysis of variance (ANOVA), followed by post hoc test with Fisher’s least significant difference, was employed for multigroup comparison. Comparison between two groups was performed with Student’s *t*-test. All *P*-values are two-tailed, and a *P*-value < 0.05 is considered significant for all statistical analysis.

## Results

### Chronic alcohol consumption upregulates hepatic GSTM1 expression in mice

**Fig. 1:**
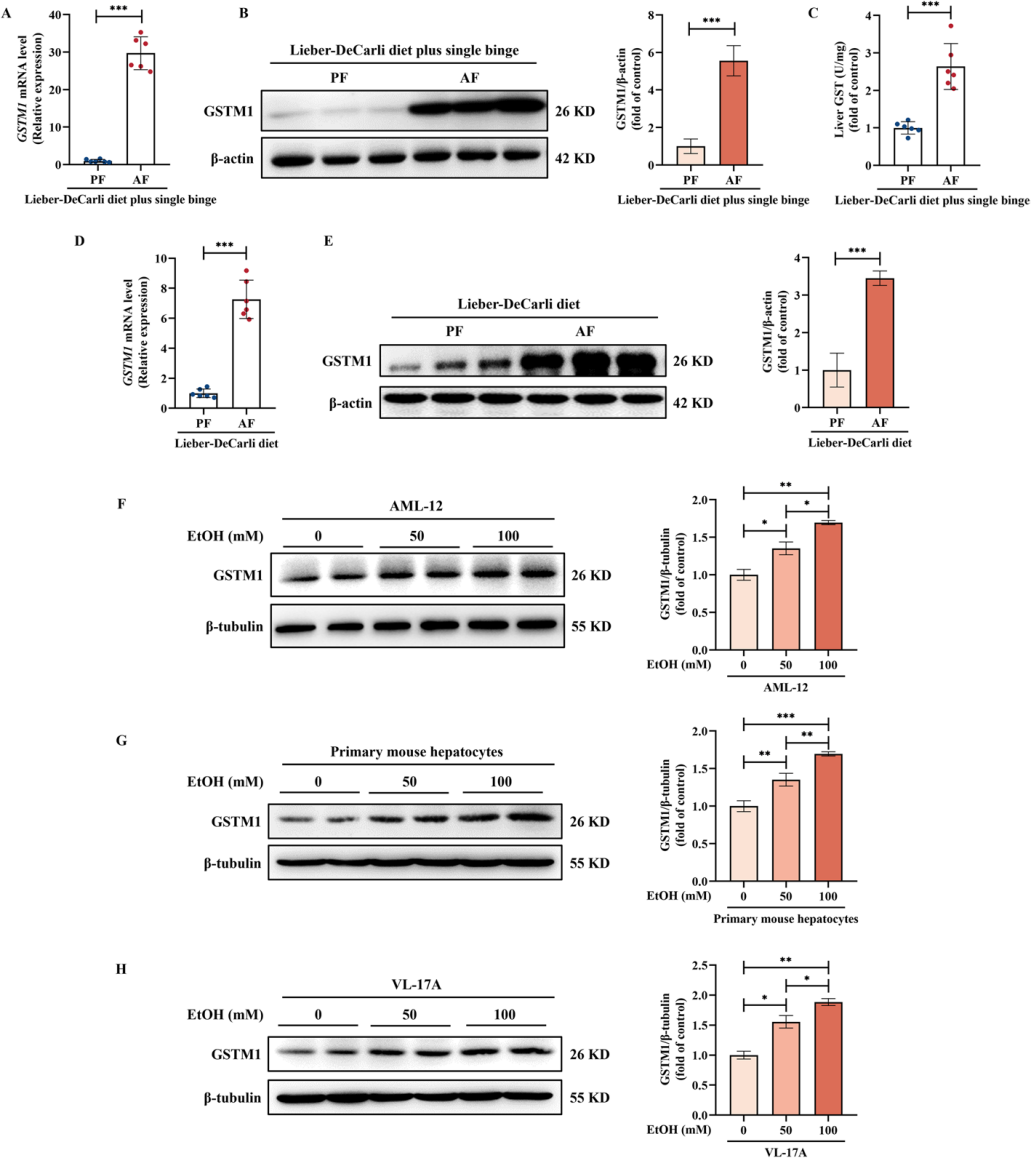
Alcohol consumption increases hepatic GSTM1 expression in ALD mice. **A**, **B** GSTM1 mRNA and protein expression, and **C** GST activity in the Lieber-DeCarli diet plus single binge model. **D**, **E** GSTM1 mRNA and protein expression in the chronic alcohol-fed model. **F**–**H** GSTM1 protein expression in mouse AML-12 hepatocytes, primary mouse hepatocytes, and human VL-17A hepatocytes. Cells were cultured with media containing ethanol for 48 h. Protein band intensity was quantified by ImageJ. Data are presented as means ± SD. Statistical comparisons were made using one-way ANOVA with Tukey’s post hoc test or one Student’s *t*-test. *P < 0.05, **P < 0.01, ***P < 0.001 versus corresponding control. *PF* pair-fed, *AF* alcohol-fed. **A**–**E** (*n* = 6); **F**–**H** (*n* = 4)

Alcohol consumption-induced impairments of hepatic antioxidant system are closely associated with the pathological process of ALD [30]. In this study, chronic plus single binge ALD mouse model was employed in Additional file 1: Fig. S1. Unexpectedly, we observed that excessive alcohol consumption indeed resulted in a significant increase of GSTM1 expression at both mRNA and protein levels in mice liver (Fig. 1A, B), concomitant with a corresponding augmentation of GST enzymatic activity (Fig. 1C). To consolidate our observations, we also examined hepatic GSTM1 expression in mice under the traditional Lieber-DeCarli ALD model (Additional file 1: Fig. S2). As shown in Fig. 1D, E, GSTM1 expression was markedly elevated in the liver of alcohol-fed (AF) mice when comparing to their pair-fed (PF) counterparts. The direct effect of ethanol on hepatocytic GSTM1 was also evaluated in cultured *AML-12* mouse hepatocytes. Ethanol exposure increased GSTM1 protein abundance in a concentration-dependent manner (Fig. 1F). Ethanol-mediated GSTM1 upregulation was also observed in mouse primary hepatocytes and human *VL-17A* hepatocytes (Fig. 1G, H).

### Hepatic GSTM1 deficiency aggravates alcohol-induced hepatic steatosis and liver injury

**Fig. 2:**
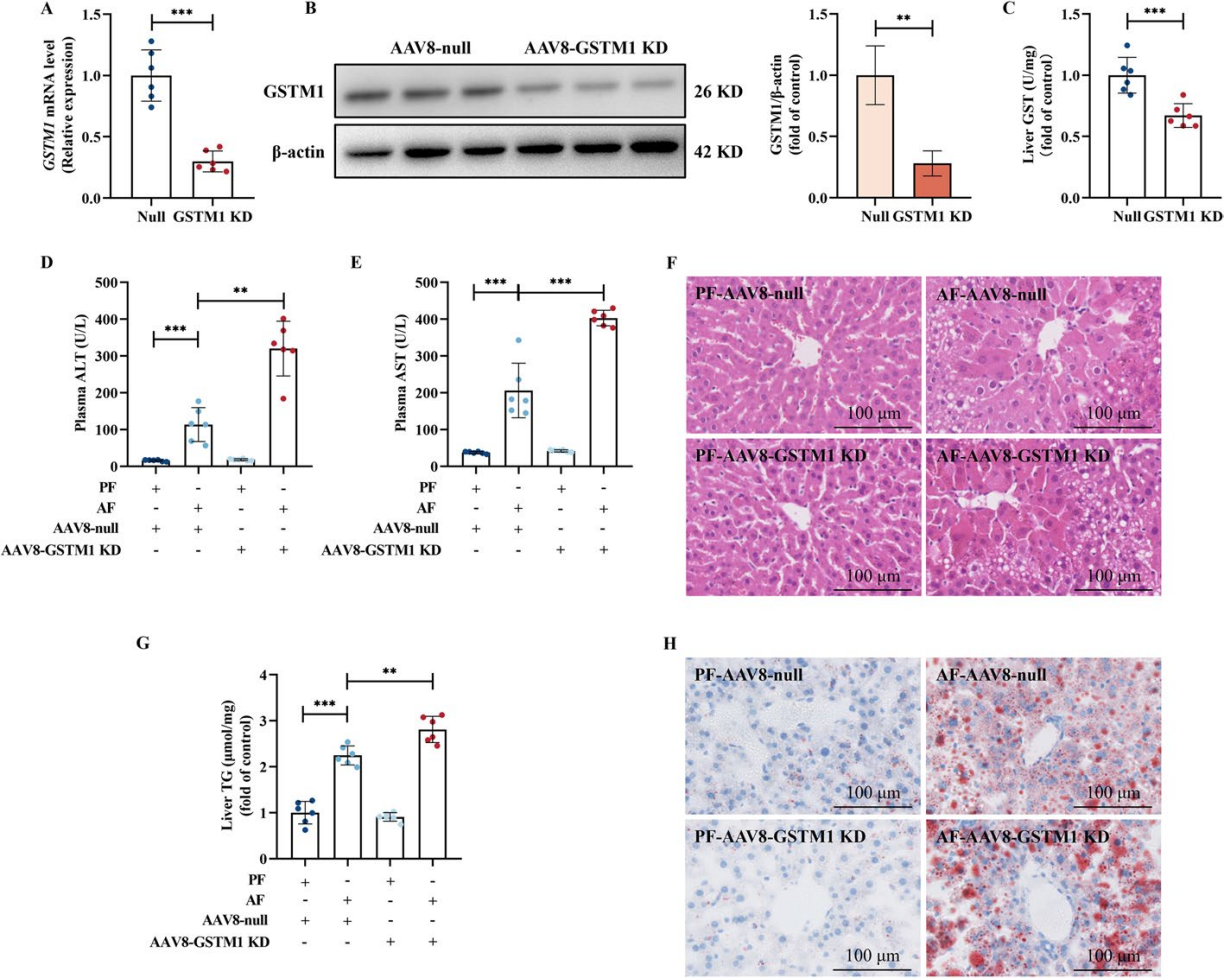
Hepatic GSTM1 loss aggravates alcohol-induced hepatic steatosis and liver injury. Hepatocyte-specific GSTM1 knockdown mice were generated by injected in the caudal vein injection with recombinant adenoassociated viral (AAV) 8 gene transfer vectors bearing a liver-specific promoter combination (TBG) with mouse GSTM1 shRNA sequence. Mice injected with null vector are served as control. **A**, **B** GSTM1 mRNA and protein expression and **C** GST activity in the hepatocyte-specific GSTM1 knockdown mice. **D**, **E** Plasma ALT and AST activities. **F** H&E staining. **G** Liver TG content. **H** Oil red O staining. Protein bands intensity was quantified by ImageJ. Data are presented as means ± SD. Statistical comparisons were made using one-way ANOVA with Tukey’s post hoc test or Student’s *t*-test. *P < 0.05, **P < 0.01, ***P < 0.001 versus corresponding control. *PF* pair-fed; *AF* alcohol-fed. **A**–**H** (*n* = 6)

To evaluate the role of hepatic GSTM1 upregulation in ALD development, the liver-specific GSTM1 knockdown mice were generated by caudal vein injection of GSTM1 shRNA delivered by AAV-8. As shown in Fig. 2A–C, over 70% of liver GSTM1 expression was effectively silenced at both mRNA and protein levels, concomitant with a 32.9% loss of GST activity. The tissue-specificity verification showed that only liver GSTM1 was downregulated among the tested tissues (Additional file 1: Fig. S3). Hepatic GSTM1 deficiency did not show a significant impact on liver function in pair-fed (PF) animals; however, it exacerbated liver injury in alcohol-fed (AF) mice, evidenced by further elevated liver enzymes (ALT and AST), as well as hematoxylin and eosin (H&E) stain (Fig. 2D–F). GSTM1 knockdown in the liver also aggravated hepatic steatosis in AF mice (Fig. 2G, H).

### Hepatic GSTM1 knockdown deteriorates alcohol-induced oxidative damage, inflammation, and early fibrosis-like alterations in liver

**Fig. 3:**
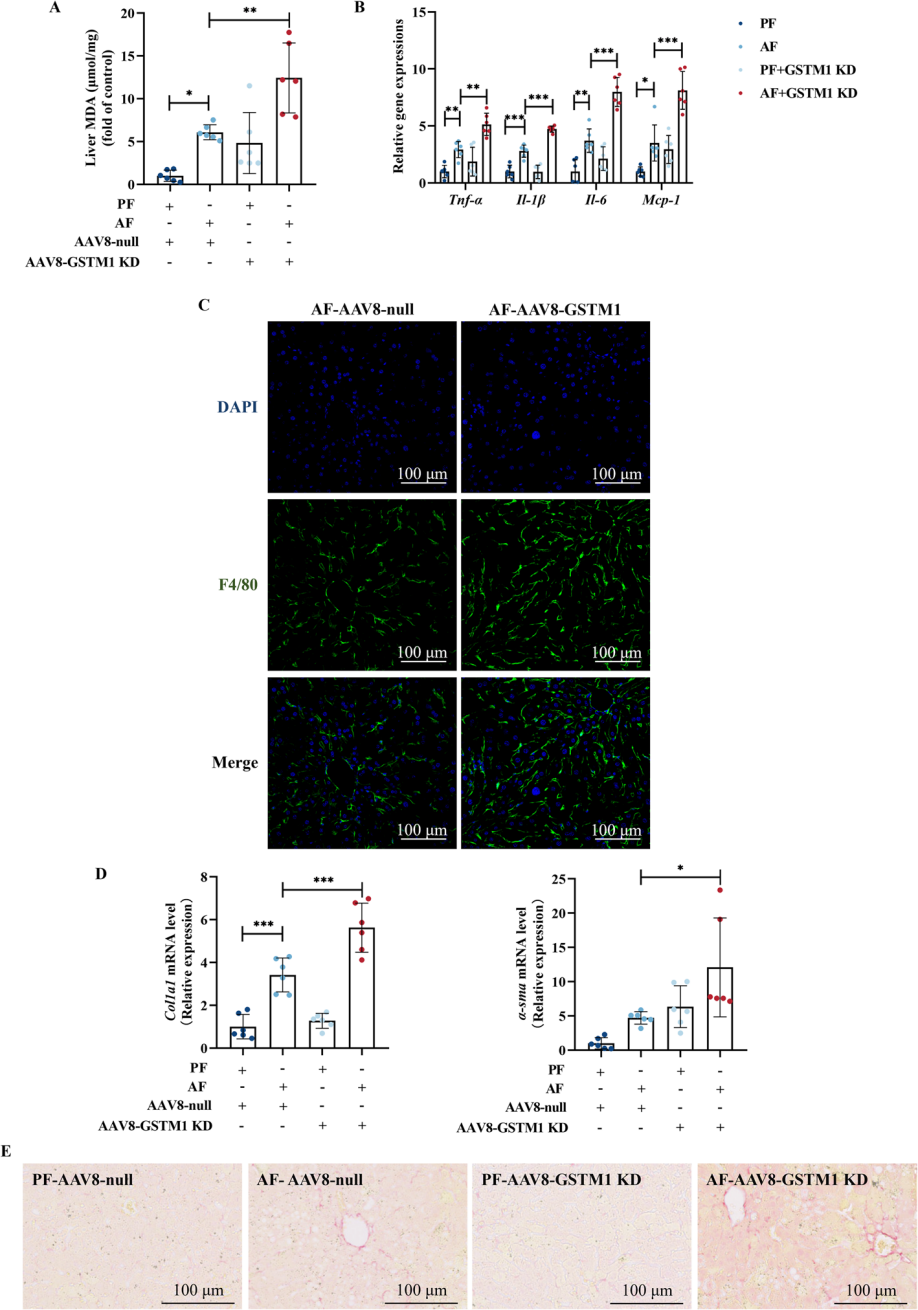
Hepatic GSTM1 knockdown deteriorates alcohol-induced oxidative damage, inflammation, and early fibrosis-like alterations in liver. **A** Liver MDA content. **B** Liver *TNF-α*, *Il-1β*, *Il-6*, and *Mcp-1* mRNA expression. **C** Immunofluorescent labeling with F4/80^+^ (green) and 4′,6-diamidino-2-phenylindole (DAPI) (blue) is shown. **D** Liver *Col1a1* and *α-sma* mRNA expression. **E** Representative liver Sirius Red staining images are shown. Data are presented as means ± SD. Statistical comparisons were made using one-way ANOVA with Tukey’s post hoc test or one Student’s *t*-test. *P < 0.05, **P < 0.01, ***P < 0.001 versus corresponding control. *PF* pair-fed, *AF* alcohol-fed. **A**–**E** (*n* = 6)

Oxidative stress, inflammation, and early fibrosis-like alterations are critical pathological hallmarks in ALD. In this study, we observe that hepatic GSTM1 loss enhanced MDA formation in the liver (Fig. 3A). Hepatic GSTM1 knockdown enhanced alcohol-induced transcriptional upregulation of proinflammatory cytokines, including *TNF-α*, *Il-1β*, *Il-6*, and *Mcp-1* (Fig. 3B), along with the infiltration of F4/80^+^ positive macrophages (Fig. 3C). In addition, early fibrosis-like alterations were also aggravated in the livers of GSTM1 knockdown mice (Fig. 3D, E).

### MiR-743a-3p downregulation contributes to alcohol-induced GSTM1 upregulation

**Fig. 4:**
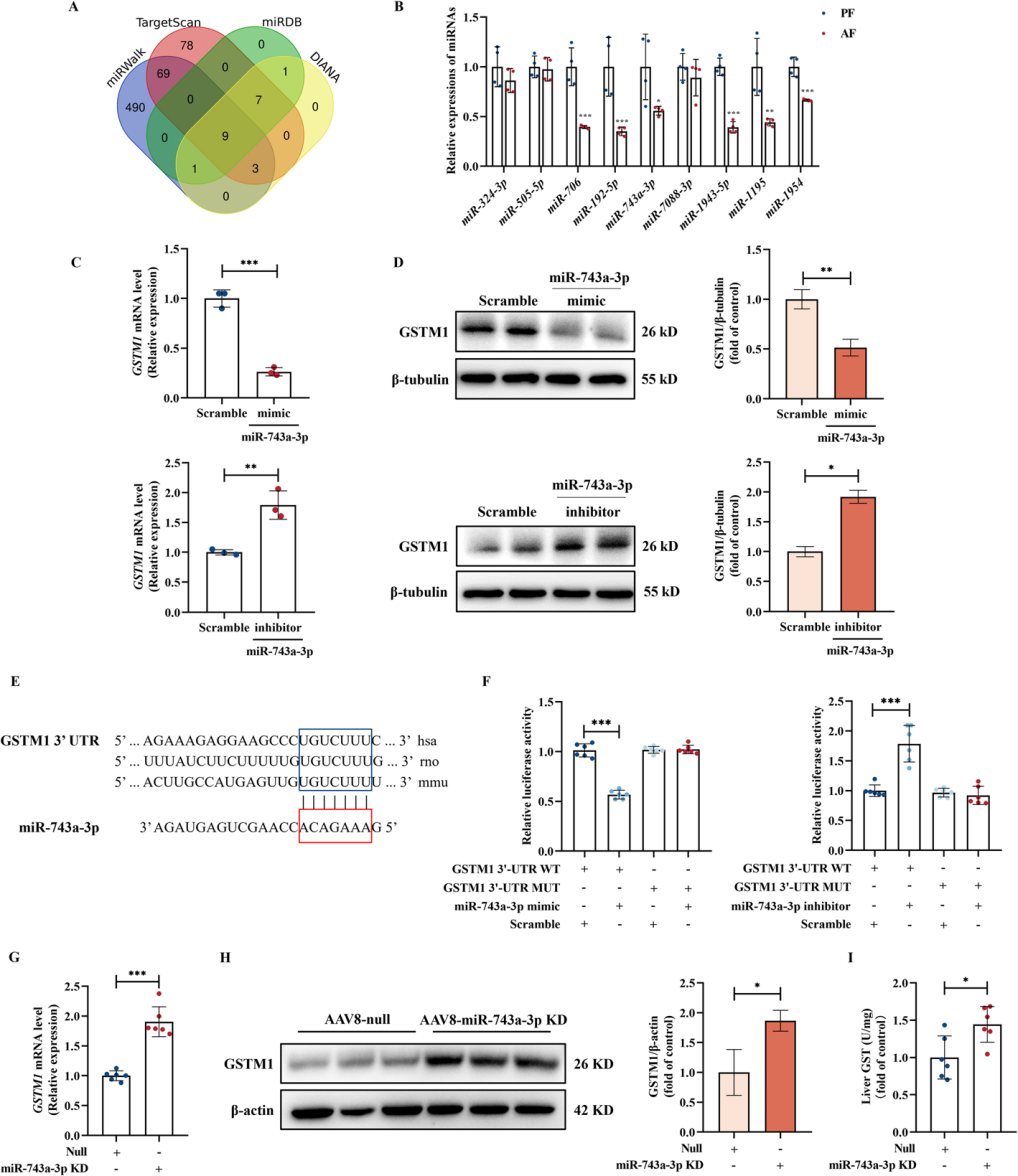
MiR-743a-3p downregulation directly contributes to alcohol-upregulated GSTM1. **A** Venn plot for the nine overlapped miRNAs. **B** Relative expression of miRNAs in the Lieber-DeCarli diet plus single binge model. **C**, **D** GSTM1 mRNA and protein expression in mouse AML-12 hepatocytes. The cells were cultured with miR-743a-3p mimic or inhibitor for 24 h. **E** Alignments of miR-743a-3p binding to the 3′-UTRs of GSTM1 mRNAs. **F** Relative luciferase activity in HEK293T cells. The cells were cotransfected with miR-743a-3p mimic or inhibitor and GSTM1-3′-UTR reporter. **G**, **H** GSTM1 mRNA and protein expression, and **I** GST activity in the hepatocyte-specific miR-743a-3p knockdown mice. Hepatocyte-specific miR-743a-3p knockdown mice were generated by injection into the caudal vein with recombinant AAV8 gene transfer vectors bearing a liver-specific promoter combination (TBG) with mouse miR-743a-3p full length sequence. Mice injected with null vector are served as control. Protein bands intensity was quantified by ImageJ. Data are presented as means ± SD. Statistical comparisons were made using one-way ANOVA with Tukey’s post hoc test or one Student’s *t*-test. *P < 0.05, **P < 0.01, ***P < 0.001 versus corresponding control. *PF* pair-fed, *AF* alcohol-fed. **B**, **D** (*n* = 4); **C** (*n* = 3); **F**–**I** (*n* = 6)

MicroRNAs (miRNAs), a class of small noncoding RNAs, are critical post-transcriptional regulators involved in ALD [31]. Here we predict miRNAs candidates targeting on GSTM1 by filtering four databases, including miRWalk (miRWalk.umm.uni-heidelberg.de), Targetscan (targetscan.org/vert_80), miRDB (miRDB—MicroRNA Target Prediction Database), and DIANA [DIANA Tools—Home (uth.gr)]. After taking the intersections, nine miRNA candidates were selected for the further test in ALD mice liver (Fig. 4A). Our data showed that six miRNAs, including miR-706, miR-192-5p, miR-743a-3p, miR-1943-5p, miR-1195, and miR-1954, were significantly downregulated in the livers of AF mice when compared with these in PF group (Fig. 4B). Using instantaneous overexpression cell model by transfecting AML-12 hepatocytes with miRNAs mimics, we observed that miR-743a-3p overexpression downregulated GSTM1 expression at both mRNA and protein levels (Fig. 4C, D, Additional file 1: Fig. S4A, B), while genetic knockdown of miR-743a-3p via transfecting hepatocytes with its antisense oligonucleotide sequence (inhibitor) robustly enhanced GSTM1 expression (Fig. 4C, D, Additional file 1: Fig. S4C). Sequence matching data indicated that the seed sequence of miR-743a-3p exhibited strong affinity with 3′-untranslated region (UTR) of GSTM1 (Fig. 4E). As shown in Fig. 4E, the miR-743a-3p recognition sites that exist in GSTM1 3′-UTR were highly conserved across different species, including human, rat, and mouse. To provide direct evidence supporting our hypothesis, the luciferase reporting system was employed and our results showed that miR-743a-3p mimic transfection reduced, while miR-743a-3p inhibitor transfection enhanced, luciferase expression from luciferase-GSTM1-3′-UTR construct in HEK293T cells, and such phenomenon was not seen when mutant structures were used (Fig. 4F). Importantly, the liver specific silencing miR-743a-3p significantly upregulated both gene and protein expressions of GSTM1 in mouse livers (Fig. 4G, H, Additional file 1: Fig. S5), accompanied with enhanced GST activity (Fig. 4I). These data collaboratively indicate that miR-743a-3p downregulation contributes to alcohol-induced GSTM1 upregulation.

### Hepatic GSTM1 deficiency abrogates the protective effects of miR-743a-3p knockdown in ALD

**Fig. 5:**
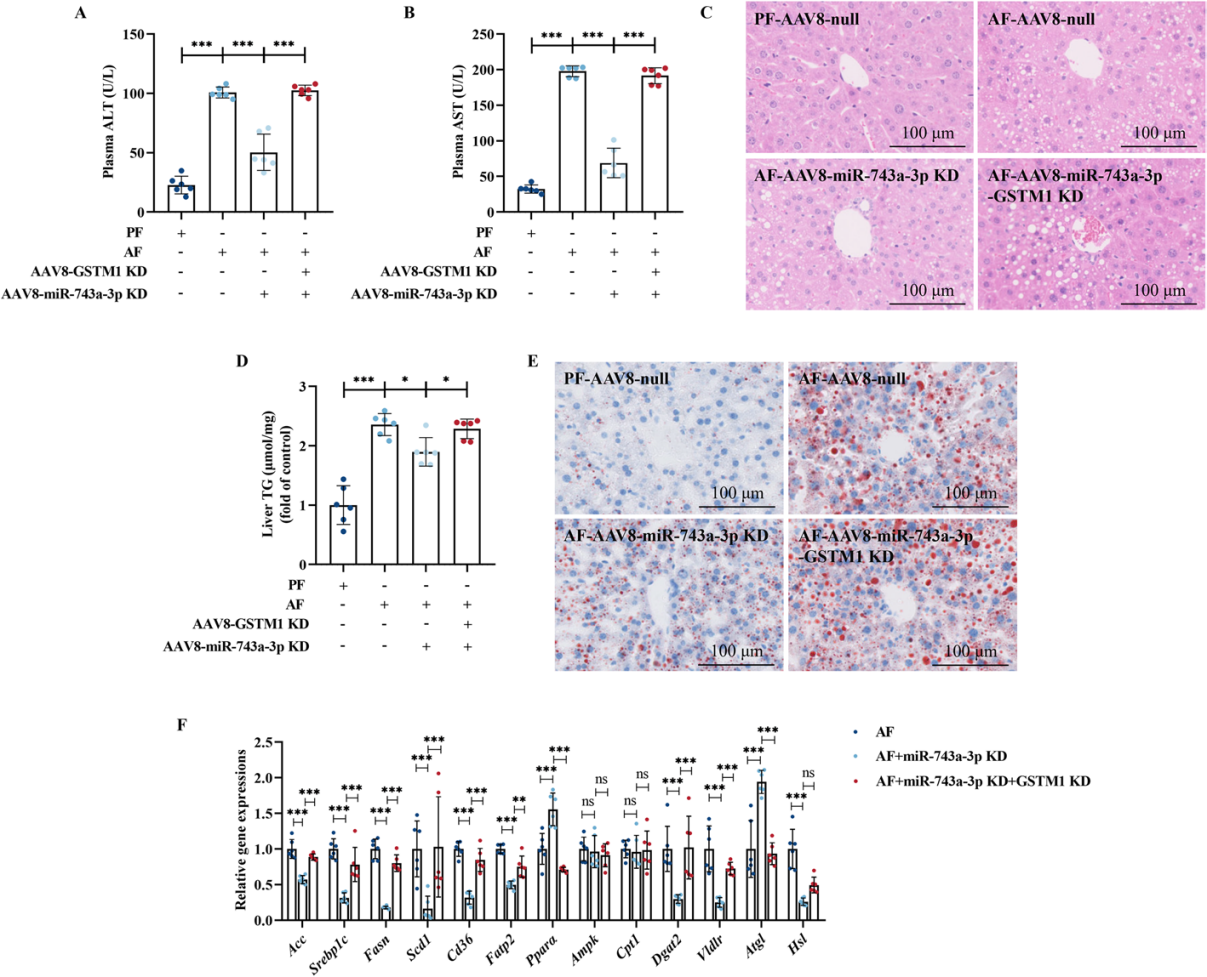
Hepatic GSTM1 loss blocks miR-743a-3p knockdown-protected ALD. Hepatocyte-specific miR-743a-3p-GSTM1 double knockdown mice were established. Mice injected with null vector are served as control. **A**, **B** Plasma ALT and AST activities. **C** H&E staining. **D** Liver TG content. **E** Oil red O staining. **F** Liver lipid metabolism genes expression. Protein band intensity was quantified by ImageJ. Data are presented as means ± SD. Statistical comparisons were made using one-way ANOVA with Tukey’s post hoc test or Student’s *t*-test. *P < 0.05, **P < 0.01, ***P < 0.001 versus corresponding control. *PF* pair-fed, *AF* alcohol-fed. **A**–**F** (*n* = 6)

**Fig. 6:**
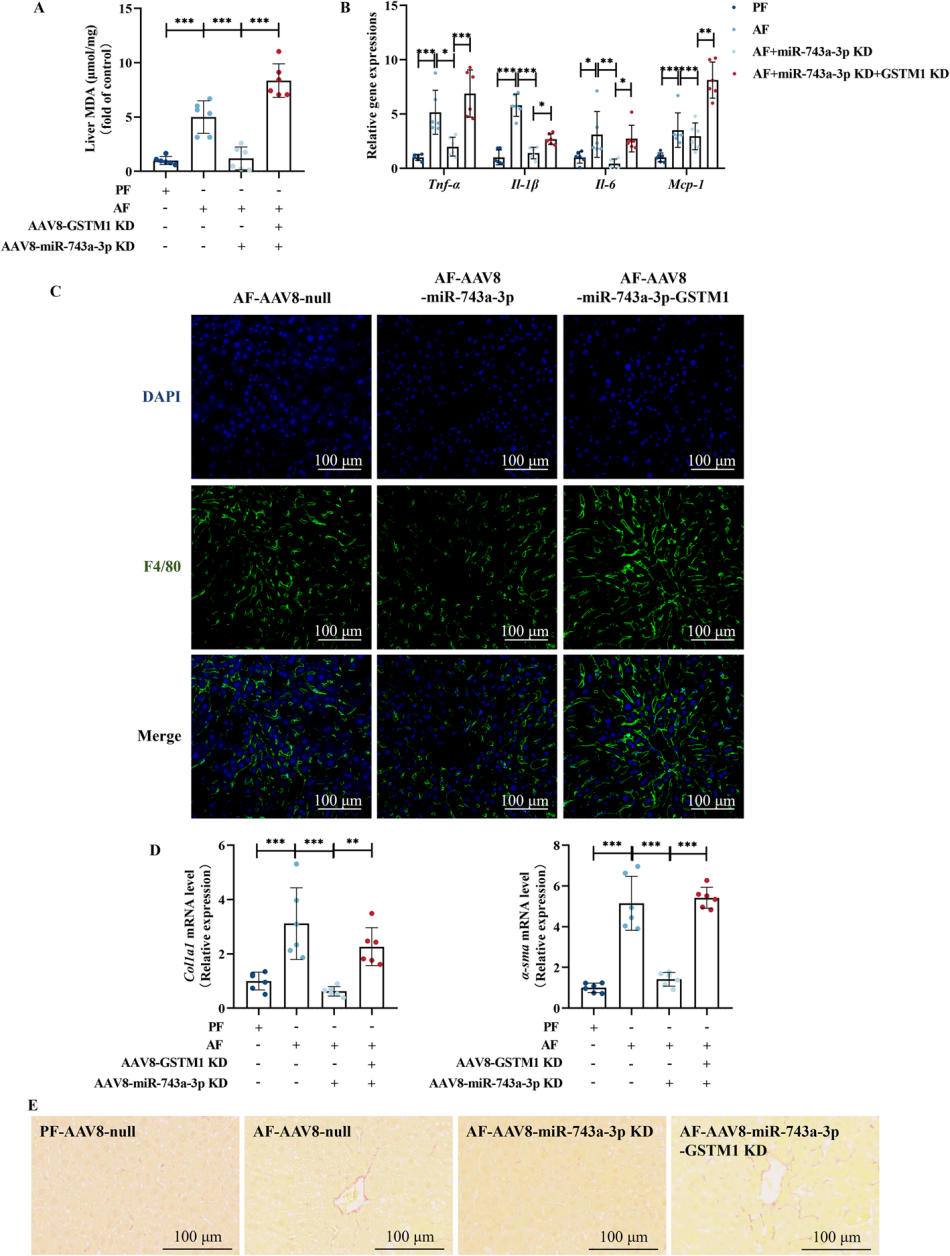
MiR-743a-3p–GSTM1 axis mediates the protective effect against alcohol-induced oxidative damage, inflammation, and early fibrosis-like alterations in liver. **A** Liver MDA content. **B** Liver *TNF-α*, *Il-1β*, *Il-6*, and *Mcp-1* mRNA expression. **C** Immunofluorescent labeling with F4/80^+^ (green) and DAPI (blue) is shown. **D** Liver *Col1a1* and *α-sma* mRNA expression. **E** Representative liver Sirius Red staining images are shown. Data are presented as means ± SD. Statistical comparisons were made using one-way ANOVA with Tukey’s post hoc test or one Student’s *t*-test. *P < 0.05, **P < 0.01, ***P < 0.001 versus corresponding control. *PF* pair-fed; *AF* alcohol-fed. **A**–**E** (*n* = 6)

We next explored the role of hepatic miR-743a-3p in chronic alcohol consumption-induced hepatic steatosis via establishing a liver-specific miR-743a-3p deficient mice model. Hepatic miR-743a-3p deficiency ameliorated alcohol-induced liver injury (Fig. 5A–C) and lessened hepatic steatosis (Fig. 5D, E). Moreover, hepatic miR-743a-3p loss improved MDA formation in the liver (Fig. 6A) and alleviated alcohol-induced gene upregulation of several proinflammatory cytokines, including *TNF-α*, *Il-1β*, *Il-6*, and *Mcp-1* (Fig. 6B), along with the reduction of F4/80^+^ positive macrophages infiltration (Fig. 6C). In addition, early fibrosis-like alterations were also ameliorated in miR-743a-3p knockdown mice liver (Fig. 6D, E). These data indicated that hepatic miR-743a-3p expression is negatively associated with alcohol-induced liver dysfunction.

To confirm the role of GSTM1 upregulation in miR-743a-3p knockdown-conferred protection against ALD, the liver-specific miR-743a-3p/GSTM1 double knockdown mice were generated and subjected to chronic alcohol feeding. As shown in Fig. 5A–E, GSTM1 deficiency in the liver abolished the protective effects of miR-743a-3p knockdown in ALD. A set of mRNA analyzation was subsequently conducted to identify the potential targets for miR-743a-3p–GSTM1-regulated lipid metabolism. Liver-specific miR-743a-3p knockdown reduced expression of genes involved in lipogenesis and lipid uptake, including *Acc*, *Srebp1c*, *Fasn*, *Scd1*, *Dgat2*, *Cd36*, *Fatp2*, and *Vldlr* and increased expression of genes for lipid catabolism, including *Pparα* and *Atgl*, which were blocked by hepatic GSTM1 silencing (Fig. 5F). Moreover, miR-743a-3p knockdown-induced alleviation of oxidative stress, inflammation, and early fibrosis-like alterations in alcohol-fed mice livers was also revoked by hepatic GSTM1 knockdown (Fig. 6). These results supported the notion that the miR-743a-3p–GSTM1 axis is a critical signal pathway in the regulation of hepatic lipid metabolism in response to alcohol challenge.

### The prevention of ASK1 activation contributes to the protective role of GSTM1 upregulation in ALD

**Fig. 7:**
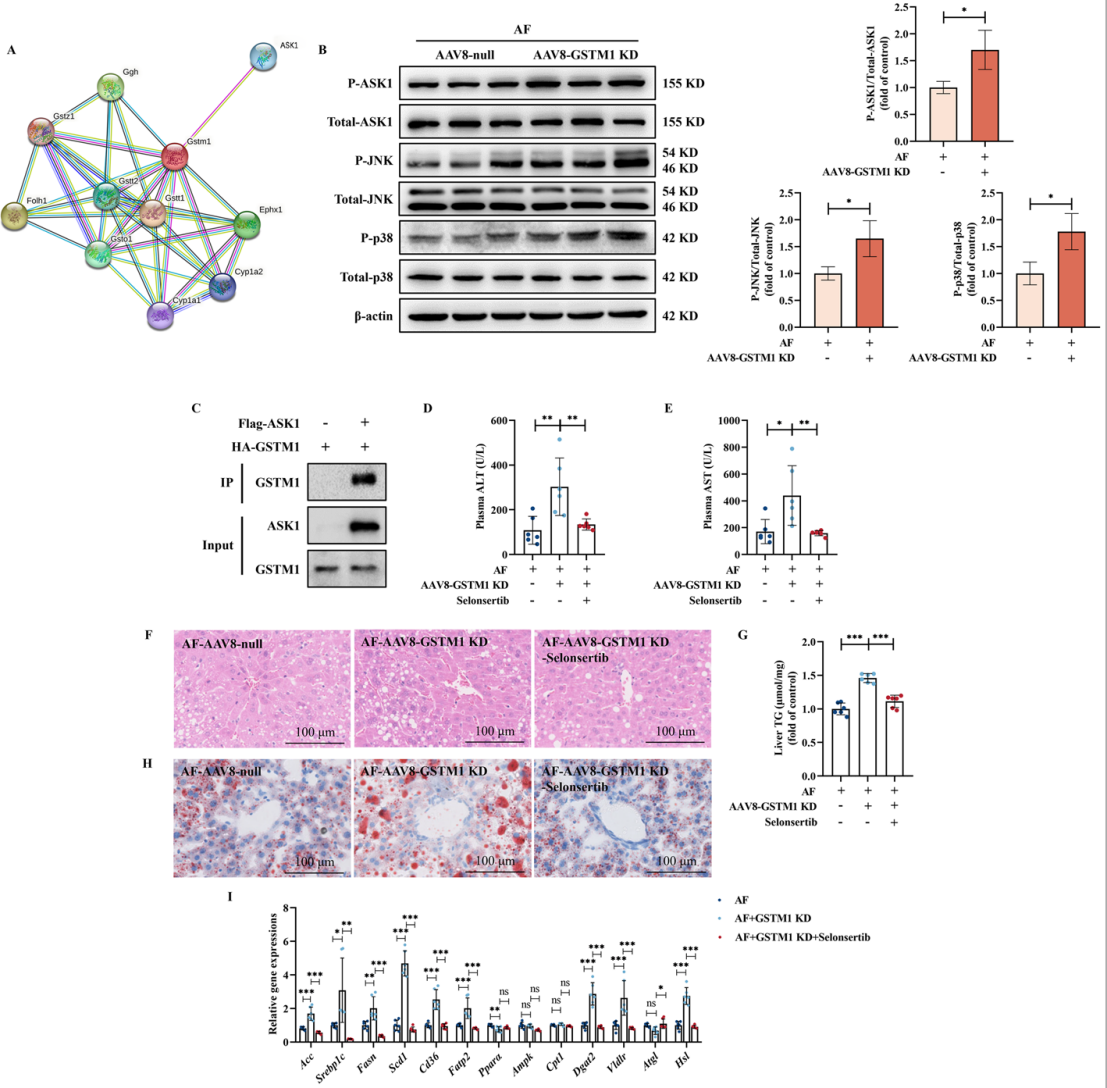
ASK1 inhibition alleviated hepatic GSTM1 knockdown-aggravated ALD. **A** Functional proteins interacting with GSTM1 predicted by STRING database. **B** The phosphorylated and total protein abundance of ASK1, JNK, and p38 in mice liver. **C** Exogenous IP assays were performed in mouse AML-12 hepatocytes transfected with HA-GSMT1 and Flag-ASK1. **D**, **E** Plasma ALT and AST activities. **F** H&E staining. **G** Liver TG content. **H** Oil red O staining. **I** Liver lipid metabolism genes expression. *AF* alcohol-fed. **B**, **D**–**I** (*n* = 6); **C** (*n* = 3)

**Fig. 8:**
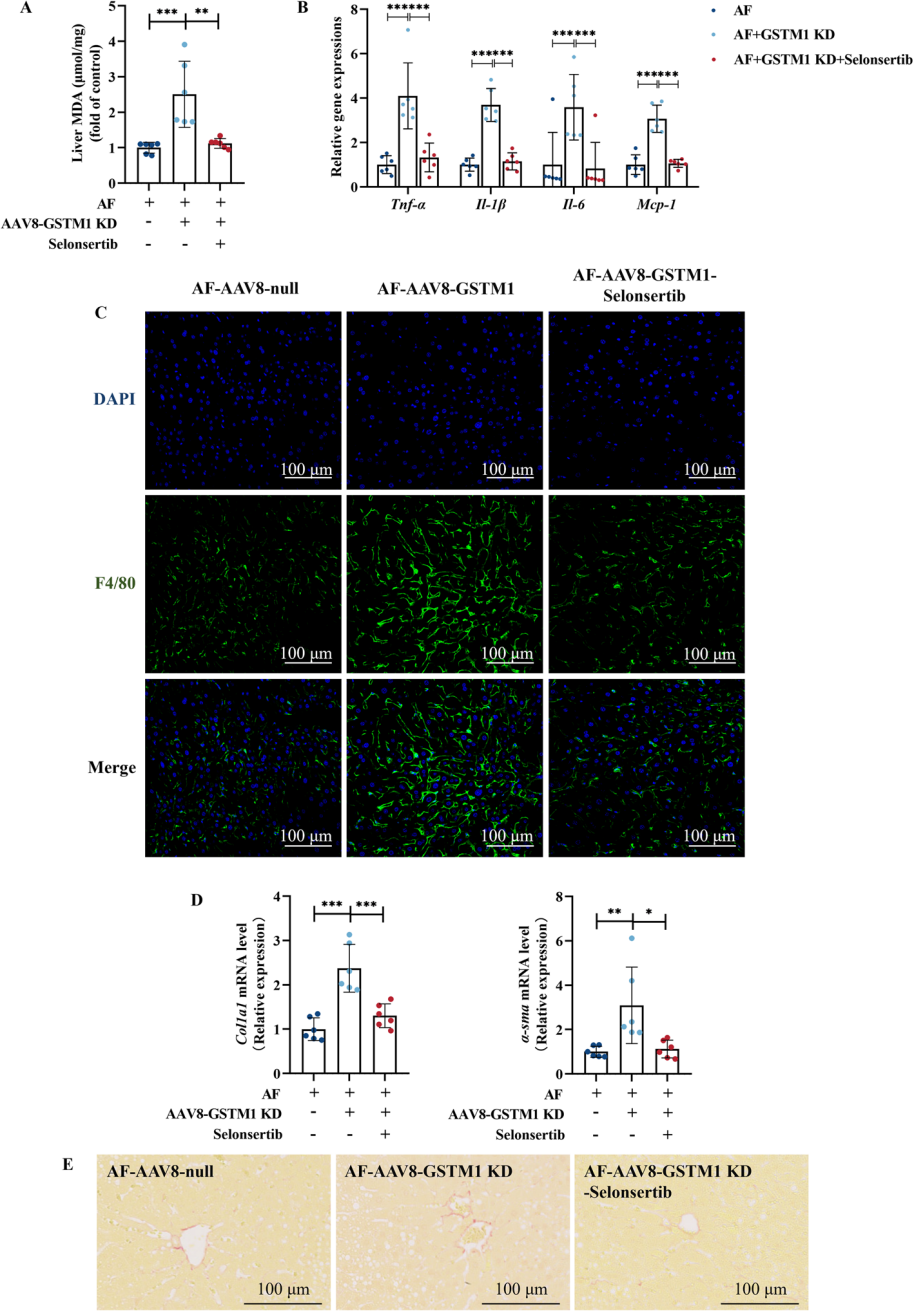
ASK1 inactivation improved hepatic GSTM1 loss-aggravated oxidative stress, inflammation, and early fibrosis-like alterations. **A** Liver MDA content. **B** Liver *TNF-α*, *Il-1β*, *Il-6*, and *Mcp-1* mRNA expression. **C** Immunofluorescent labeling with F4/80^+^ (green) and DAPI (blue) is shown. **D** Liver *Col1a1* and *α-sma* mRNA expression. **E** Representative liver Sirius Red staining images are shown. Data are presented as means ± SD. Statistical comparisons were made using one-way ANOVA with Tukey’s post hoc test or one Student’s *t*-test. *P < 0.05, **P < 0.01, ***P < 0.001 versus corresponding control. *PF* pair-fed, *AF* alcohol-fed. **A**–**E** (*n* = 6)

We next asked the reason through which hepatic GSTM1 upregulation protects against ALD development. The STRING database was performed to predict the functional proteins interacting with GSTM1. As shown in Fig. 7A, ten genes were predicted to have the potential to bind to GSTM1. Among these genes, ASK1 activation has been reported to contributed to the physiological progress of ALD [32]. We posited that ASK1 was a potential downstream target to regulate GSTM1 deficiency-associated ALD aggravation. To test the hypothesis, the activity of ASK1 was first analyzed in the liver samples of mice with liver-specific GSTM1 knockdown, and an increased phosphorylation of ASK1, JNK, and p38 was observed upon chronic alcohol consumption (Fig. 7B). The exogenous co-IP detection showed GSTM1 bound with ASK1 (Fig. 7C), indicating that GSTM1 can interact with ASK1, suppressing its phosphorylation activity and subsequent downstream JNK/p38 signaling pathway activation. To confirm the role of ASK1 activation in GSTM1 deficiency-related aggravation of ALD, selonsertib, a specific pharmacological inhibitor of ASK1, was selected to block ASK1 activity in experimental animals. ASK1 inhibition rescued GSTM1 deficiency-aggravated liver injury and hepatic steatosis in mice subjected to chronic alcohol exposure (Fig. 7D–H). The dysregulated expression of genes involved in lipid metabolism by GSTM1 knockdown was largely rescued by ASK1 inhibition (Fig. 7I). Additionally, aggravated oxidative stress, inflammation, and early fibrosis-like alterations resulting from hepatic GSTM1 loss were improved by ASK1 inhibition (Fig. 8A–E).

## Discussion

This study provides initial evidence that chronic alcohol consumption induces hepatic GSTM1 upregulation as a result of miR-743a-3p downregulation. Hepatic GSTM1 deficiency aggravates hepatic fat accumulation and liver injury in the setting of chronic alcohol exposure. The liver-specific knockdown of miR-743a-3p leads to GSTM1 upregulation and alleviates liver pathologies of ALD and the beneficial effect of miR-743a-3p knockdown in ALD is abolished by GSTM1 deficiency. Further mechanistic investigations unravel that the activation of ASK1-JNK/p38 signaling contributes to the detrimental effects of GSTM1 deficiency in ALD. Taken together, our data suggest that exploring translational pathways to enhance GSTM1 expression may be a promising strategy for preventing and treating ALD.

Hepatic steatosis is a classic pathological hallmark in the early-stage of ALD and oxidative stress contributes to the abnormal expression in lipid metabolic genes and further excessive fat accumulation in the liver, which is accompanied with mild injury and inflammation and early fibrosis-like alteration [39]. In this study, we observed that hepatic GSTM1 knockdown aggravated fatty liver and liver injury in response to chronic alcohol exposure. These data indicated that hepatic GSTM1 upregulation functioned as a protective mechanism against alcohol-related fatty liver disease. In support of this notion, a recent investigation has reported that depleting GSTM2, an isoenzyme of GSTM1, exacerbated hepatic steatosis and liver injury in non-alcoholic steatohepatitis mice [11]. It has been well-documented that GSTM1 loss aggravated oxidative stress and inflammation in various diseases [40–43]. Consistence with these findings, we provided initial evidence that the existence of GSTM1 is required to resist alcohol-stimulated oxidative stress, inflammation, and fibrosis-like alteration in the liver.

GSTM1, also known as GST1, and GTM1, is a member of GSTs superfamily, which belongs to phase II detoxification enzymes. As a highly frequent polymorphisms gene, GSTM1 is closely associated with the development of disease in multiple organs, such as cardiovascular disease, pulmonary disease, renal disease, and liver disease [16, 33–35]. As the most highly expressed subtype of GSTM in the liver, GSTM1 null genotype is positively associated with drug-induced liver injury, hepatitis, liver cirrhosis, and even hepatocellular carcinoma [8]. GSTM1 null polymorphism also enhanced the incidence risk of ALD [20, 21]. However, there are not published data that have addressed how chronic alcohol exposure regulates GSTM1 in liver, as well as whether and how GSTM1 expression and activity contribute to the pathogenesis of ALD. Existing evidence has reported the adaptive increase of GSTM1 expression in the liver against drug-induced oxidative stress and endoplasmic reticulum stress [36, 37], which are the common pathological features in ALD. In line with these findings, we observed that GSTM1 expression was robustly upregulated in both parenchymal hepatocytes of alcohol-fed mice and cultured hepatocytes. Accordingly, we found that GSTs activity was significantly elevated in chronic alcohol-fed mice liver, consistent with that in patients with ALD [38].

Besides hepatic parenchymal cells, the activation of hepatogenic immune cells, such as Kupffer cells, monocyte-derived macrophages [44], and natural killer cells [45], contributes to the pathological progress of ALD. The accumulation of macrophages has been observed in the liver of patients with ALD [46]. Existing evidence suggested that alcohol-disrupted intestinal barrier, followed by harmful gut microbiota or its metabolites (e.g. lipopolysaccharide) transporting into the liver via circulation, leads to the activation of immune cells in liver [47]. Activated immune cells induce abnormal lipid metabolism by producing proinflammatory factors acting on hepatic parenchyma, which further promoting the pathological process of ALD [48]. In this study, we observed that hepatic GSTM1 knockdown aggravated F4/80^+^-positive macrophages accumulation, along with the transcriptional activation of proinflammatory factors, implying a potentially important role of liver macrophage on hepatic GSTM1 knockdown-accelerated hepatic steatosis and liver injury. In support of our observation, similar phenomena have also been found in lots of hepatic gene knockout/knockdown ALD models studies [49, 50].

Since GSTM1 was transcriptionally regulated by chronic alcohol consumption, to explore the mechanism(s) underlying alcohol-induced GSTM1 upregulation, transcription factors were initially considered. After literature filtrating, we found that nuclear factor erythroid 2-associated factor 2 (Nrf2) has been identified as a transcription factor regulating GSTM1 expression [51]. However, the existing evidence including ours showed that chronic alcohol consumption decreased Nrf2 activity [27, 52], which was inconsistent with alcohol-induced mRNA increase of GSTM1. Therefore, the involvement Nrf2-regulated GSTM1 was excluded. Changes in liver miRNA expression are one of the important pathological mechanisms of ALD [31]. It has been reported that GSTM1 can be regulated by miR-423-5p and miR-3188 in various types of cells [53–55]. However, limited studies have reported the involvement of miRNAs in the regulation of GSTM1 in ALD. Here we identifiy miR-743a-3p as a direct and negative regulator of GSTM1 in ALD based on the following findings: (1) miR-743a-3p was downregulated in alcohol-exposed mice liver, (2) miR-743a-3p could bind with 3′-UTR region of GSTM1 and (3) genetically knocking-down miR-743a-3p increased GSTM1 expression in both mice liver and hepatocytes, while miR-743a-3p overexpression inhibited GSTM1 expression. These data demonstrated that miR-743a-3p is the upstream target for alcohol-regulated GSTM1. It has been reported that oxidative stress, a pathological hallmark in ALD, decreased miR-743a expression in mouse hippocampal cell line [56]; however, whether and how oxidative stress induces miR-743a-3p reduction in ALD mice liver was not investigated in our study, which is a limitation of this study.

Very few studies have reported the biological function of miR-743a-3p in health maintaining. Previous studies revealed that miR-743a-3p participated in anticancer drug floxuridine-induced mouse mammary carcinoma apoptosis and suppressed the proliferation of metanephric mesenchymal cells [57, 58]. To the best of our knowledge, no published data have addressed the role of miR-743a-3p in ALD progression before our investigation. In the present study, we reported for the first time that hepatic miR-743a-3p is a novel target to prevent ALD. Similar to GSTM1, alcohol-induced decrease in miR-743a-3p represents a protective mechanism for ALD development as knocking down miR-743a-3p significantly reversed alcohol-induced pathological changes in ALD mice liver. Importantly, we identified GSTM1 as a direct target of miR-743a-3p and upregulation of GSTM1 contributes to the protective effects of miR-743a-3p knockdown against ALD. These findings implied that targeting on the miR-743a-3p–GSTM1 axis, via either reducing miR-743a-3p or increasing GSTM1 expressions are promising ways for alcohol-induced fatty liver prevention. It is worth to mention that our study only tested the beneficial role of miR-743a-3p–GSTM1 against the early stage of ALD due to the limitation of Lieber-DeCarli model, which only induces mild steatosis and liver injury [59]. Since hepatic steatosis is not the primary pathological risk in patients with advanced ALD with hepatitis, fibrosis, or cirrhosis, further studies are still needed to disclose the effect of miR-743a-3p–GSTM1 axis on other advanced stages of ALD via employing corresponding animal models.

The potential mechanism underlining GSTM1-regulated pathological progression of ALD was further explored in this study. In addition to catalyzing the conjugation of GSH to xenobiotics, emerging evidence revealed that GSTM1 can regulate the activity of other enzymes by directly binding to them, such as adenosine 5′-monophosphate-activated protein kinase and TANK binding kinase 1 [60, 61]. In this study, protein docking analysis predicted that GSTM1 can interact with ASK1, and this was confirmed by both our exogenous co-IP experiment and previous investigations [62]. ASK1 is a MAPK kinase family member that plays a critical role in stress-induced damage by activating the JNK/p38 signaling cascades [63]. It has been well-documented that chronic alcohol consumption led to a significant increased phosphorylation of ASK1, JNK, and p38, which in tune stimulated the ASK1-JNK/p38 pathway and disturbed lipid metabolism related genes expression [64]. Either genetic depletion or pharmacological inhibition of ASK1 reversed alcohol-induced hepatic steatosis in mice [32], implying that antagonizing the activity of ASK1 is a potential strategy for treating ALD. Here we identify ASK1 as a main downstream target in GSTM1-regulated ALD based on the following evidence: (1) phosphorylated-ASK1 was significantly elevated in hepatic GSTM1 knockdown mice liver, (2) GSTM1 loss increased the JNK/p38 pathway activation, and (3) ASK1 inhibition abolished GSTM1 deficiency-induced liver dysfunction, along with the improvement of lipid metabolism related genes disorders in ALD mice. Although how GSTM1 interacts with ASK1 was not investigated in this study, existing evidence has showed that the C-terminal portion of GSTM1 and the N-terminal region of ASK1 were crucial for their binding [62]. Additionally, GSTM2 has been recently reported to combine with the N-terminal portion of ASK1 and, therefore, blocking its activity [11]. A seemingly contradictory observation in our study is that increased GSTM1 was coexisted with ASK1 activation in chronic alcohol-fed mice liver. This could be explained by the facts that ASK1 phosphorylation can also be stimulated by multiple signal pathways, including cellular repressor of E1A stimulated genes 1, and receptor-interaction protein kinase 3, etc., under chronic alcohol exposure [64, 65], while alcohol-induced feedback increase of GSTM1 is not strong enough to completely neutralize ASK1 activation stimulated by these kinases. However, additional increase in GSTM1 expression by liver specific knockdown of miR-743a-3p improved hepatic steatosis and liver injury in ALD mice, implying that GSTM1 is a promising therapeutic target for ALD.

The abnormal enhancement of lipolysis in adipocytes has been reported to be implicated in alcoholic hepatic steatosis [66]. Whether lipolysis stimulation contributes to hepatic GSTM1 knockdown-aggravated hepatic steatosis was evaluated in this study. We observed that fat weight and fat weight to body weight ratio were reduced in alcohol-fed mice, along with increased circulatory free fatty acid level (Additional file 1: Fig. S6). Nevertheless, hepatic GSTM1 knockdown did not enhance alcohol-stimulated lipolysis in adipose tissue (Additional file 1: Fig. S6). These data excluded the participation of lipolysis in GSTM1-regulated ALD.

We report for the first time that liver GSTM1 is required to protect against ALD. Liver GSTM1 loss aggravated the pathologies via promoting ASK1-JNK/p38 signaling activation. Further mechanistic investigations indicated that miR-743a-3p is a direct and negative upstream regulator of GSTM1 in the liver. Our findings demonstrate that the strategies targeting on the miR-743a-3p–GSTM1 pathway represent a potential therapeutic choice for the treatment of ALD.

### Supplementary Information

**Additional file 1.** Materials and methods and supplementary figure.

## Data Availability

All data and materials are available.

## Abbreviations

ALD: Alcohol-related liver disease
ASK1: Apoptosis signal-regulating kinase 1
AAV: Adenoassociated viral
ALT: Alanine aminotransferase
AST: Aspartate aminotransferase
Acc: Acetyl-CoA carboxylase
Ampk: Adenosine 5′-monophosphate-activated protein kinase
Atgl: Adipose triglyceride lipase
α-sma: α-Smooth muscle actin
Cd36: Cluster of differentiation 36
Cpt1: Carnitine palmitoyltransferase 1
Col1ɑ1: Collagen type I alpha 1
Dgat2: Diacylglycerol acyltransferase 2
Fasn: Fatty acid synthase
Fatp2: Fatty acid transport protein 2
GSTs: Glutathione *S*-transferases
GSTM1: Glutathione *S* transferase mu 1
HCC: Hepatocellular carcinoma
Il-1β: Interleukin 1beta
Il-6: Interleukin 6
IP: Immunoprecipitation
JNK: C-Jun N-terminal kinase
MDA: Malonaldehyde
miRNA: MicroRNA
Mcp-1: Monocyte chemoattractant protein 1
Nrf2: Nuclear factor erythroid 2-associated factor 2
Pparα: Peroxisome proliferator-activated receptor alpha
Srebp1c: Sterol regulatory element-binding proteins 1c
Scd1: Stearoyl-CoA desaturase 1
SD: Standard deviation
TG: Triglyceride
Tnf-α: Tumor necrosis factor alpha
UTR: Untranslated region
Vldlr: Very low-density lipoprotein receptor
