# Supplementary material for: The micro-743a-3p–GSTM1 pathway is an endogenous protective mechanism against alcohol-related liver disease in mice
Source: Cell Mol Biol Lett. 2024 Mar 12;29:35. doi: 10.1186/s11658-024-00557-x (PMC10936097; doi:10.1186/s11658-024-00557-x)
Supplement: Supplementary file 1 — Additional file 1. Materials and methods and supplementary figure. [file 11658_2024_557_MOESM1_ESM.docx]

**Additional file 1**

**The micro-743a-3p-GSTM1 pathway is an endogenous protective mechanism against alcohol-related liver disease in mice**

Tiantian Xu^1, #^, Yan Pan^2, #^, Qinchao Ding^1, #^, Feiwei Cao^1^, Kaixin Chang^2^, Jiannan Qiu^2^, Hui Zhuge^2^, Liuyi Hao^1^, Haibin Wei ^2^, Caijuan Si^3^, Xiaobing Dou^2^, Songtao Li^1, 3^

^1^ School of Public Health, Zhejiang Chinese Medical University, Hangzhou, Zhejiang, PR. China

^2^ School of Life Science, Zhejiang Chinese Medical University, Hangzhou, Zhejiang, PR. China

^3^ Department of Clinical Nutrition, Affiliated Zhejiang Hospital, School of Medicine, Zhejiang University, Hangzhou, Zhejiang, PR. China

^#^ These authors contribute equally to this paper.

*To whom correspondence should be addressed E-mail: [lisongtao@zcmu.edu.cn](mailto:lisongtao@zcmu.edu.cn) (S. Li).

**Contents list**

Additional Materials and Methods……………………………………………. 3

Fig. S1……………………………………………………………….10

Fig. S2……………………………………………………………….11

Fig. S3……………………………………………………………….12

Fig. S4……………………………………………………………….13

Fig. S5……………………………………………………………….14

Fig. S6……………………………………………………………….15

Table S1………………………………………………………….… 16

Table S2……………………………………………….…………… 18

Table S3……………………………………………….…………… 19

Additional References………………………………………………...………... 20

**Additional materials and methods**

**Chemicals**

Ethanol was purchased from Sigma-Aldrich (St. Louis, MO); Selonsertib was obtained from MedChemExpress (Shanghai, China).

**Animals**

All the animal procedures were approved by the Institutional Animal Care and Use Committee of Zhejiang Chinese Medical University, and the animals were maintained according to the guidelines of the Animal Experimental Center of Zhejiang Chinese Medical University (approval number: 20220221-23). Eight-week-old male *C57BL/6J* mice were purchased from Shanghai SLAC Laboratory Animal Co. Ltd (Shanghai, China, License No. SCXK (Hu) 2017-0005). All mice were housed on a 12-h light-dark cycle at 23 ± 2℃ with 55 ± 5% relative humidity. All the interventions were performed after one week of environmental acclimatization. Food intake and body weight were recorded daily and weekly, respectively. At the end of the experiments, mice were sacrificed under pentobarbital solution (30 mg per kg body weight, ip.). Plasma and tissue samples were collected for further analysis.

The Lieber-DeCarli Alcohol-related liver disease (ALD) model was established as described previously ^[1]^. Mice were fed with the Lieber-DeCarli alcohol liquid diet (alcohol-fed; AF) or isocaloric maltose dextrin control liquid diet (pair-fed; PF) for four weeks. There are two groups in total (n = 6 mice/group) and animals were fed for 4 weeks. In the first week, mice were given a modified Lieber-DeCarli liquid diet (Trophic Animal Feed High-Tech Co., Ltd. Nantong, China) without alcohol for adaption to the liquid diet for 3 days, then the alcohol-fed group was given with Lieber-DeCarli liquid diet containing 1% ethanol for 2 days, 2% ethanol for 2 days where the ethanol content (%, v/v) in the diet was 4% for the second week and was gradually increased by 1% every week, and reaching 6% in the last week. The amount of food given to the pair-fed mice was the same as the alcohol-fed mice consumed the previous day.

Lieber-DeCarli plus single binge ALD model was established based on the Lieber-DeCarli ALD model. The mice were randomly divided into PF and AF groups (n = 6 mice/group). After 4-week Lieber-DeCarli alcohol liquid diet feeding, AF mice were administered one dose of ethanol (4 g/kg) by gavage, and PF mice were given isocaloric maltose dextrin. Samples were collected 4 h after the gavage.

Liver-specific glutathione S transferase mu 1 (GSTM1) knockdown mice and corresponding control mice were generated by caudal vein injection with recombinant adeno-associated viral serotype 8 (AAV8) gene transfer vectors containing a hepatocyte-specific thyroxine-binding globulin (TBG) promoter combination with either mouse GSTM1 shRNA sequence (AAV8-GSTM1 KD) or empty as vector control (AAV8-null). The core sequence of shGSTM1 was 5’-TTCTCCACAATGTCTGCAC-3'. There are four groups in total (n = 6 mice/group), and animals were fed for 4 weeks. AAV8 vectors were administered by tail vein injection at a dose of 1×10^12^ viral titer/mL in a total volume of 100 μL/mouse one week before alcohol-fed. Recombinant AAV8 vectors encoding shGSTM1 under the control of TBG (a liver-specific promoter) were generated by HanBio Technology Co. Ltd. (Shanghai, China).

Liver-specific microRNA (miR)-743a-3p knockdown mice and corresponding control mice were generated by caudal vein injection with recombinant AAV8 gene transfer vectors containing a hepatocyte-specific TBG promoter combination with either mouse miR-743a-3p whole length sequence (AAV8-miR-743a-3p KD) or empty as vector control (AAV8-null). The core sequence of shmiR-743a-3p was 5’-GAAAGACACCAAGCUGAGUAGA-3'. There are four groups in total (n = 6 mice/group), and animals were fed for 4 weeks. AAV8 vectors were administered by tail vein injection at a dose of 1×10^12^ viral titer/mL in a total volume of 100 μL/mouse one week before alcohol-fed. Recombinant AAV8 vectors encoding shmiR-743a-3p under the control of TBG were generated by HanBio Technology Co. Ltd. (Shanghai, China).

Liver-specific miR-743a-3p-GSTM1 double knockdown mice were randomly divided into PF, AF, AF + miR-743a-3p KD, and AF + miR-743a-3p KD + GSTM1 KD (n = 6 mice/group). ALD model was established by feeding mice with the Lieber-DeCarli alcohol liquid diet for 4 weeks. After 4-week Lieber-DeCarli alcohol liquid diet feeding, AF mice were administered one dose of ethanol (4 g/kg) by gavage. AAV8-miR-743a-3p KD vectors were administered by tail vein injection at 1×10^12^ viral titer/mL in a total volume of 100 μL/mouse two weeks before alcohol-fed. AAV8-GSTM1 KD vectors were administered by tail vein injection at a dose of 1×10^12^ viral titer/mL in a total volume of 100 μL/mouse one week before alcohol-fed.

Selonsertib intervention: Mice were randomly divided into AF, AF + GSTM1 KD, and AF + GSTM1 KD + Selonsertib intervention (n = 6 mice/group). ALD model was established by feeding mice with the Lieber-DeCarli alcohol liquid diet for 4 weeks. After 4-week Lieber-DeCarli alcohol liquid diet feeding, AF mice were administered one dose of ethanol (4 g/kg) by gavage. Selonsertib was administrated by gavage at 30 mg/kg body weight/two days.

**Immunohistochemistry**

For hematoxylin and eosin (H&E) and Sirius red staining, liver tissue samples were fixed in a 4% paraformaldehyde solution and further embedded in paraffin. Liver sections (4 μm) were deparaffinized in xylene and rehydrated through decreasing ethanol concentrations. H&E and Sirius red staining were performed using a staining kit (G-Clone, Beijing, China), respectively. For Oil red O staining, liver tissues were embedded in the Tissue-Tek OCT compound (Sakura, Tokyo, Japan). Frozen sections, 8-μm thick, were subjected to Oil red O staining according to the instructions of the Oil red O staining kit (Solarbio, Beijing, China). Images were captured by Zeiss Axio Observer A1 inverted microscope (Oberkochen, Germany).

**Immunofluorescence staining**

Fresh liver tissues were OCT-embedded. Frozen sectioned and blocked with PBS containing 5% BSA for 60 min. The samples were then incubated with F4/80 antibody (1:100 dilution, Santa Cruz Biotechnology, CA) incubation overnight at 4 ℃. Then, the samples were washed twice with PBS containing 0.1% Tween-20 and incubated with green fluorophore-conjugated secondary antibody (1: 500 dilution, Beyotime, Shanghai, China) for 1 h at room temperature. Samples were then stained with 1 μg/mL DAPI (Beyotime, Shanghai, China) for 10 min. Images were taken by the laser scanning confocal microscope (Zeiss, Jena, German, LSM880).

**Plasma analysis**

Blood samples from the inferior vena cava were collected and centrifuged at 4℃ and 3000 rpm for 15 min. The supernatants were collected for the measurements of alanine transaminase (ALT), aspartate transaminase (AST), and free fatty acid (FFA) using commercial assay kits (Nanjing Jiancheng Bio Co., Nanjing, China).

**Triglyceride (TG) content assay**

Liver TG content was measured by a commercial TG assay kit (Abcam, Cambridge, UK), according to the manufacturer's recommended protocol.

**Malondialdehyde (MDA) content assay**

Liver MDA content was measured by a commercial malonaldehyde assay kit (Beyotime, Shanghai, China), according to the manufacturer's recommended protocol.

**GST activity**

GST activity in liver samples were measured using a Glutathione S-transferase activity assay kit (Solarbio, Beijing, China), according to the manufacturer's recommended protocol.

**Cell culture**

Primary mouse hepatocytes were isolated as reported previously ^[2,3]^, Briefly, mice were anesthetized with pentobarbital (30 mg per kg body weight, ip.). Livers were firstly perfusion with ice Hank's Balanced Salt Solution (HBSS, Monad, Wuhan, China) via the portal vein and followed by digested for 15 min at 37℃ in digestion buffer (RPMI-1640 containing 1% fetal bovine serum (FBS, Biological Industries, ISR), 0.1 mg/mL DNase-I (Sigma-Aldrich, St. Louis, MO), 0.2 mg/mL Collagenase IV (Sigma-Aldrich, St. Louis, MO), and 0.8 mg/mL Dispase II (Sigma-Aldrich, St. Louis, MO). After digestion, dissociated cells were collected and filtered through a 100 µm cell strainer (BD Biosciences, San Jose, CA) then followed by centrifuge at 50 g for 3 min at 4℃. Pellets were suspended with 20 mL 40% ice-cold percoll (GE Healthcare, Bensalem, PA) and centrifuged at 180 g for 7 min at 4℃. After this step, primary hepatocytes were transferred to matrigel-covered plate and cultured with Dulbecco's Modified Eagle Medium/Ham's Nutrient Mixture F-12, 1:1 (DMEM/F-12, Hyclone, Logan, UT) containing 10% (v/v) FBS (CellMax, Beijing, China), 100 U/mL penicillin (Solarbio, Beijing, China), 100 U/mL streptomycin (Solarbio, Beijing, China) at 37°C in a humidified atmosphere of 5% CO_2_ and 95% air.

*AML-12*, a non-transformed mouse hepatocyte cell line was obtained from American Type Culture Collection (ATCC, Manassas, VA). *AML-12* cells were cultured in DMEM/F-12 containing 10% (v/v) FBS, 5 mg/mL insulin (Solarbio, Beijing, China), 5 μg/mL transferrin (Solarbio, Beijing, China), 5 ng/mL selenium (Sigma-Aldrich, St. Louis, MO), 40 ng/ml dexamethasone (Solarbio, Beijing, China), 100 U/mL penicillin, 100 U/mL streptomycin, at 37°C in a humidified atmosphere of 5% CO_2_ and 95% air.

*VL-17A*, a HepG2-based reformed cell line, was conducted according to previous study ^[4]^. HepG2 cell line was obtained from Cell bank of Chinese Academy of Sciences (Shanghai, China). HepG2 cells were stably transfected with both cytochrome P450 2E1 (CYP2E1) and alcohol dehydrogenase (ADH) to construct *VL-17A* cell line. *VL-17A* cells were cultured in DMEM (Gibco, Waltham, MA) supplemented with 10% FBS and 100 U/mL penicillin/streptomycin at 37°C in a humidified atmosphere of 5% CO_2_ and 95% air.

*HEK293T* cells were purchased from ATCC (Manassas, VA). HEK293T cells were cultured in DMEM (Hyclone, Logan, Utah) medium containing 10% (v/v) FBS, 100 U/mL penicillin, and 100 U/mL streptomycin, at 37°C in a humidified atmosphere of 5% CO_2_ and 95% air.

**RNA interference**

Cultured cells were transfected with miRNA mimic, or miR-743a-3p inhibitor (GenePharma, Shanghai, China) using siRNA-mate (GenePharma) according to the manufacturer's instructions. In the control group, cells were transfected with scramble siRNA (GenePharma).

**GSTM1 3′-UTR luciferase assay**

### The plasmid containing the Luc-GSTM1-3′-untranslated region (UTR) construct (Product ID: C09009) was used in reporter assays purchased from GenePharma (Shanghai, China). A mutation of the Luc-GSTM1-3’-UTR mutant was done by replacing the sequence of miR-743a-3p binding region from 5’-atgcggctctgtgctccagacctgaGCTCctGCTGTGTCcTTCccaatgtgcagggcctcaagaagatct-3' to 5’-atgcggctctgtgctccagacctgaCGAGctCGACACAGcAAGccaatgtgcagggcctcaagaagatct-3' (uppercases indicate mutation). The plasmid contains firefly luciferase fused to the 3’-UTR of human GSTM1 and Renilla luciferase that functions as a tracking gene. Luciferase activity assays were performed according to manufacturer protocols. Briefly, HEK293T cells were seeded in 12-well plates, and co-transfected with miR-743a-3p mimic/inhibitor and GSTM1-3’-UTR reporter. Cells were harvested following transfection for 48 h. Firefly and Renilla luciferase activities were measured sequentially with the dual luciferase assay kit (GenePharma). The activities were normalized with Renilla luciferase activities and expressed in relative luciferase activity units.

### Co-immunoprecipitation

The co-immunoprecipitation (co-IP) assay was performed as described before ^[5]^. In brief, cells were lysed in co-IP buffer (20 mM Tris, pH 7.5, 150 mM NaCl, 1% Triton X-100, and 1 mM EDTA) containing protease inhibitors (ComWin Biotech, Beijing, China) on ice for 30 min. Then, the cells were centrifuged, and the supernatant was collected, followed by incubation with Flag Magnetic Beads (Sigma-Aldrich, St. Louis, MO) with gentle rocking overnight at 4 ℃. The next day, the mixture was pelleted, washed three times with cold co-IP buffer, and then analyzed by western blot.

**Quantitative real-time polymerase chain reaction (qRT-PCR)**

Total RNA was isolated from the tested tissues or cultured cells using Trizol reagent (Invitrogen, Carlsbad, CA). Extracted RNA was then transcribed into cDNA by reverse transcription reagent kit (Monad, Wuhan, China) according to the manufacturer's protocol. The primers were synthesized by Sangon biotech co. ltd (Shanghai, China), and primer sequences were shown in Table S1. The data were normalized to 18s rRNA or U6 mRNA level and presented as fold changes.

**Western blot analysis**

Western blots were performed as described previously ^[2]^. In brief, liver tissues or cultured cells were lysed with RIPA buffer (Boster Biological Technology, Wuhan, China) supplemented with protease and phosphatase inhibitors (Sigma-Aldrich, St. Louis, MO). The protein samples were loaded into SDS-PAGE gels and transferred to PVDF membranes (Millipore, Bedford, MA). The membranes were blocked with 5% fat-free milk in TBST (Servicebio, Wuhan, China) and then reacted with primary antibodies at 4℃ for 12-16 h. After washing with TBST, the membranes were incubated with secondary antibodies for 1 h at room temperature. Finally, the immunoreactivity of protein expression was visualized with electrogenerated chemiluminescence kit (Vazyme, Nanjing, China). The immunoblots were quantified by measuring the density of each band with Image-J Software. The antibodies were listed in Table S2.

**Additional figures**

**Fig. S1**


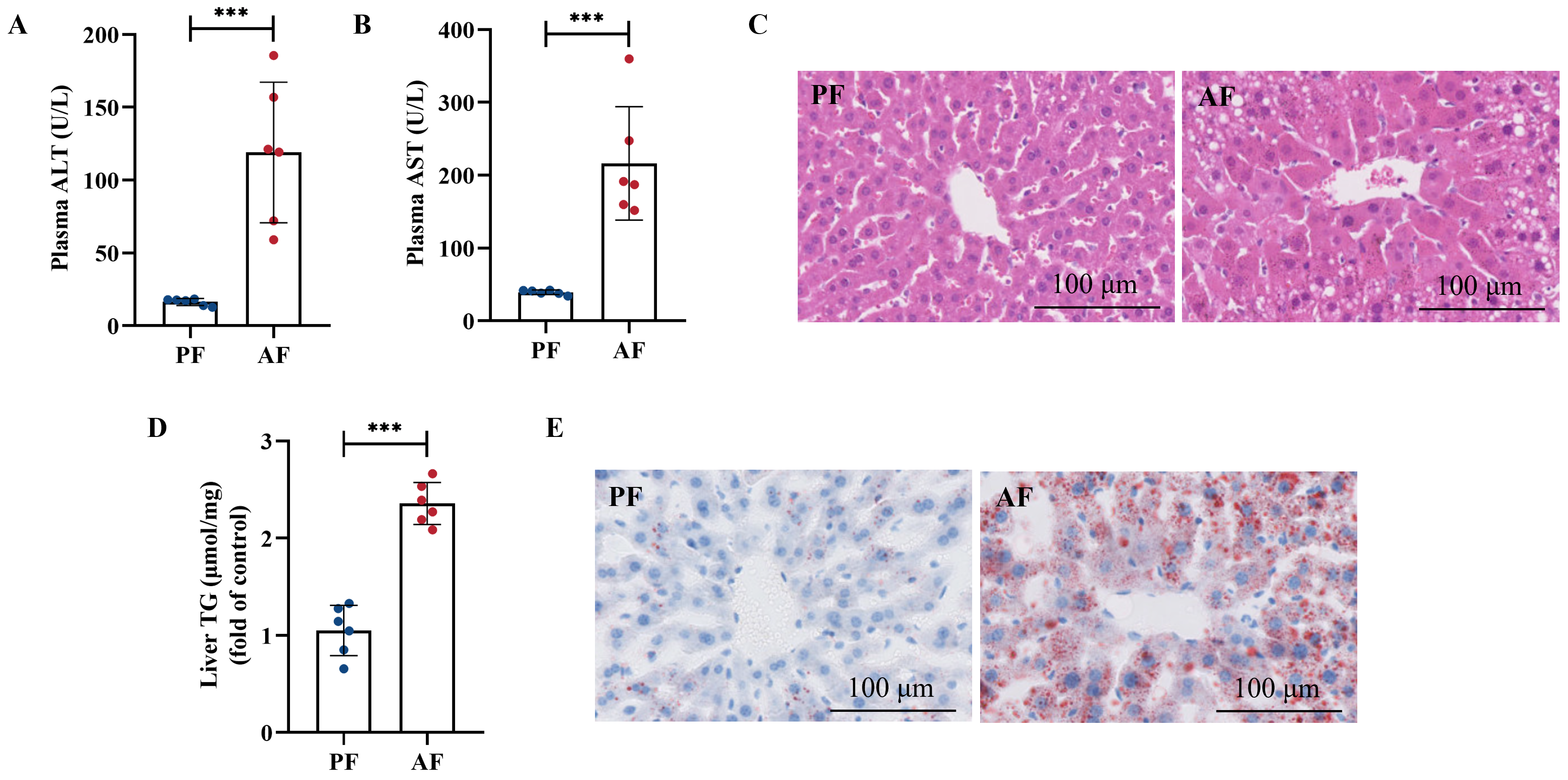


Fig. S1. ALD model was successfully established by Lieber-DeCarli diet plus single binge. (A) Plasma ALT activities. (B) Plasma AST activities. (C) H&E staining. (D) Liver TG content. (E) Oil red O staining. Data are presented as means ± SD. **p* <0.05, ***p* <0.01, ****p* <0.001 vs. corresponding control. PF, pair-fed; AF, alcohol-fed. A-E (n = 6).

**Fig. S2**


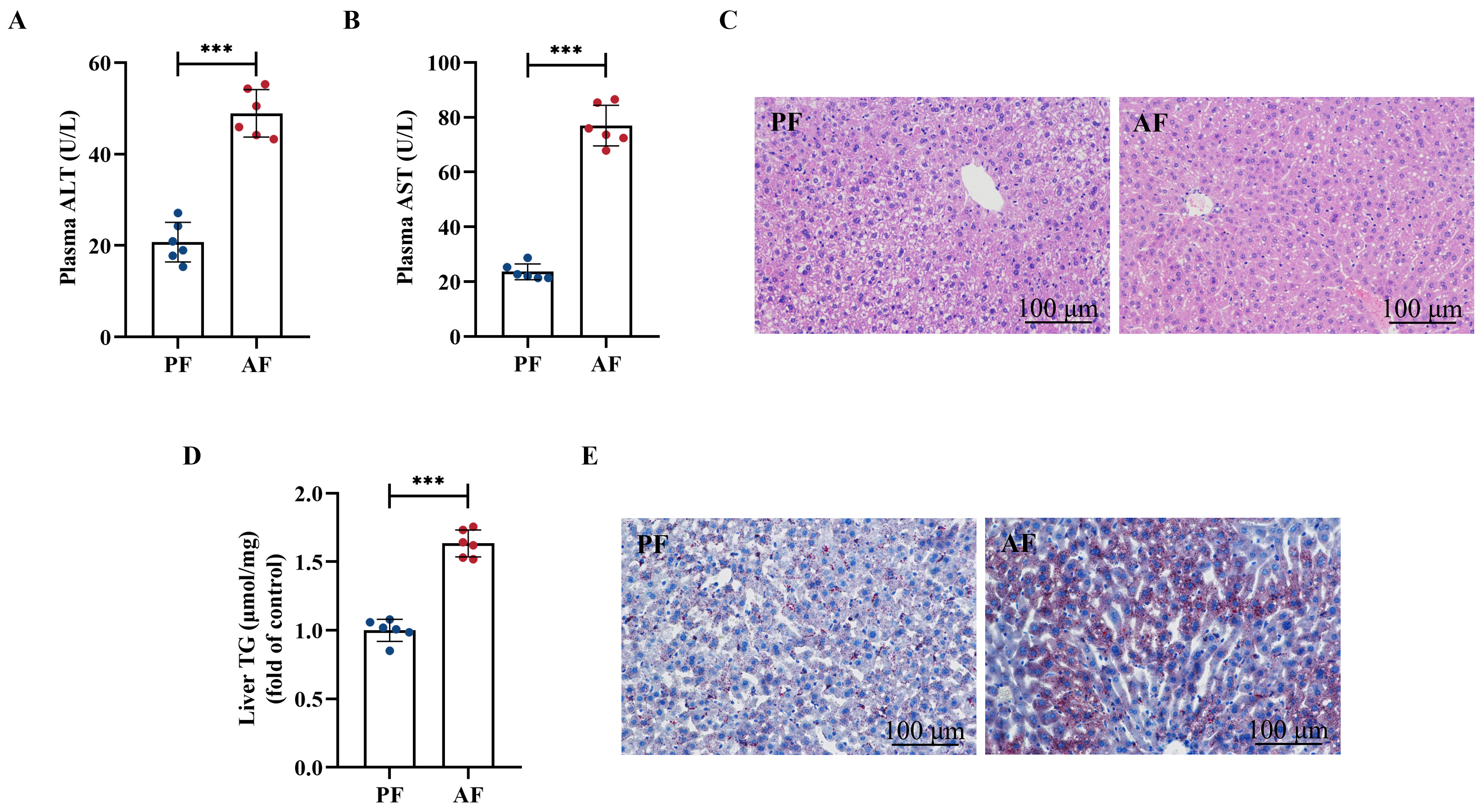


Fig. S2. A traditional ALD model was successfully established by Lieber-DeCarli diet. (A) Plasma ALT activities. (B) Plasma AST activities. (C) H&E staining. (D) Liver TG content. (E) Oil red O staining. Data are presented as means ± SD. **p* <0.05, ***p* <0.01, ****p* <0.001 vs. corresponding control. PF, pair-fed; AF, alcohol-fed. A-E (n = 6).

**Fig. S3**


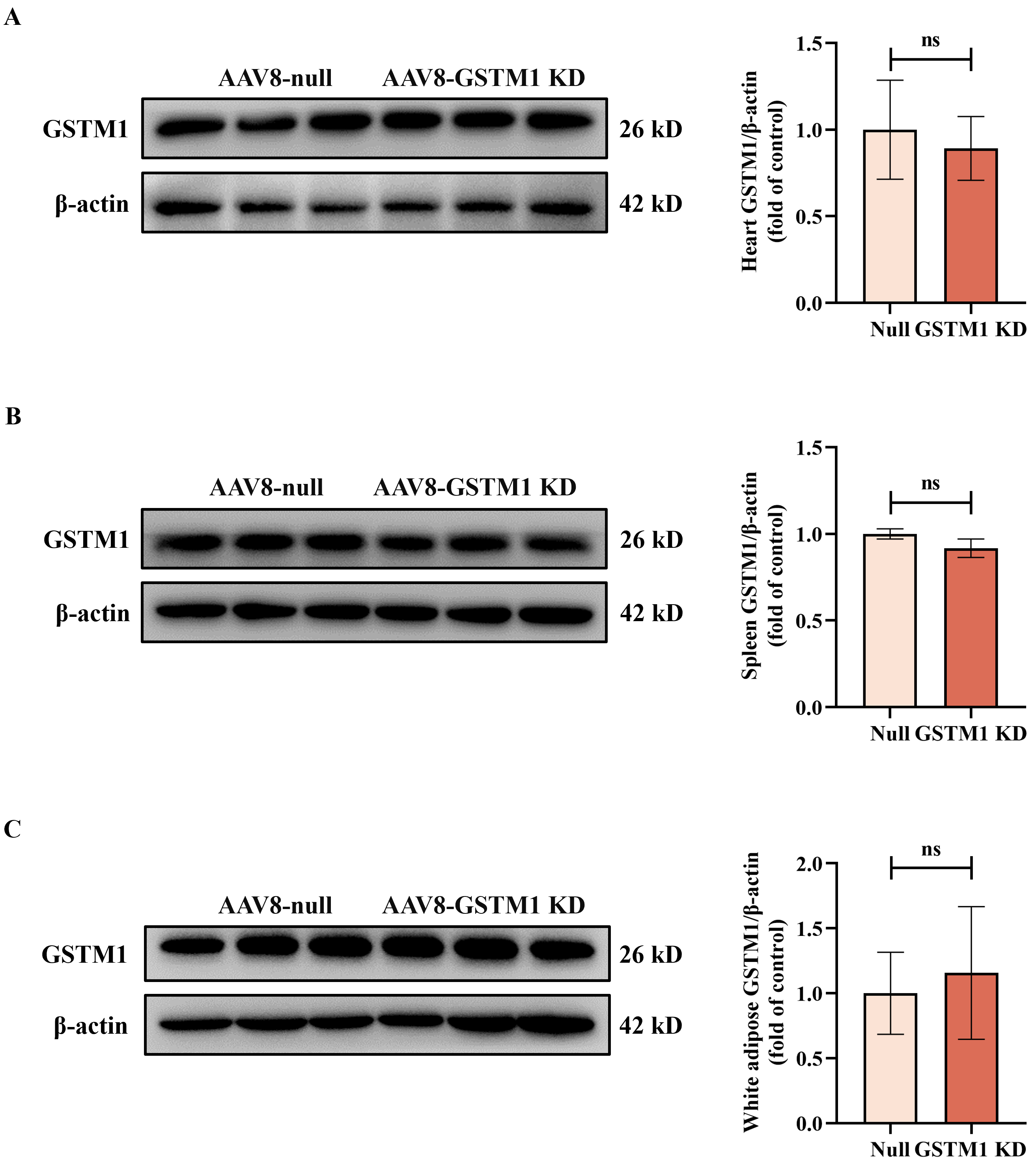


Fig. S3. GSTM1 protein expression was detected in different tissues of liver-specific GSTM1 knockdown mice. Total lysates from different tissues were subjected to Western blot assay for GSTM1. Protein bands intensity were quantified by ImageJ. Data are presented as means ± SD. ns represents no statistical difference. A-D (n = 3).

**Fig. S4**


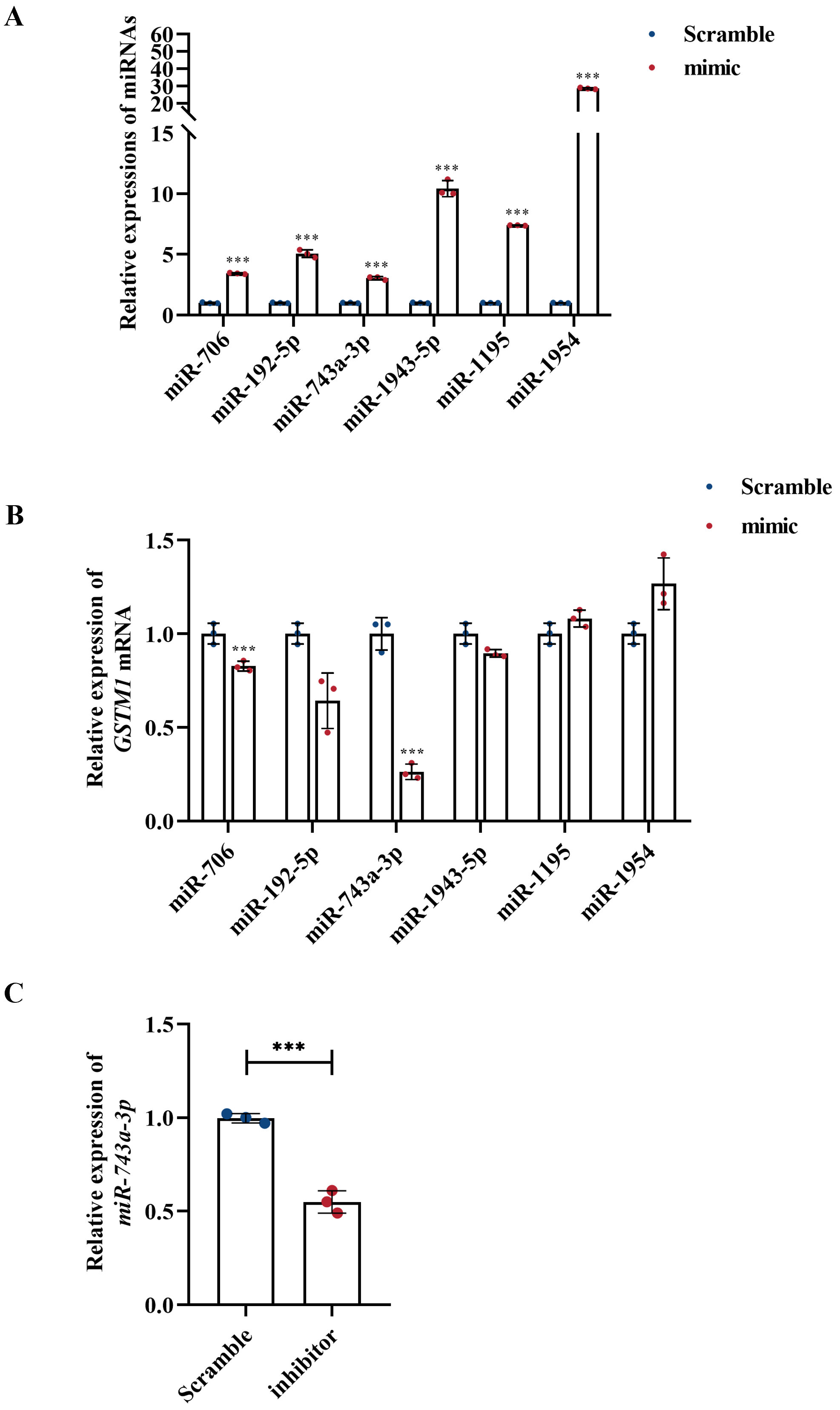


Fig. S4. Relative expression of *miRNAs* and *GSTM1* was assayed in mouse *AML-12* hepatocytes. Cells were transfected with mimic or inhibitor for 24 h. (A) Relative expression of *miRNAs*. (B) *GSTM1* mRNA level. (C) Relative expression of *miR-743a-3p*. Data are presented as means ± SD. **p* <0.05, ***p* <0.01, ****p* <0.001 vs. corresponding control. A-C (n = 3).

**Fig. S5**


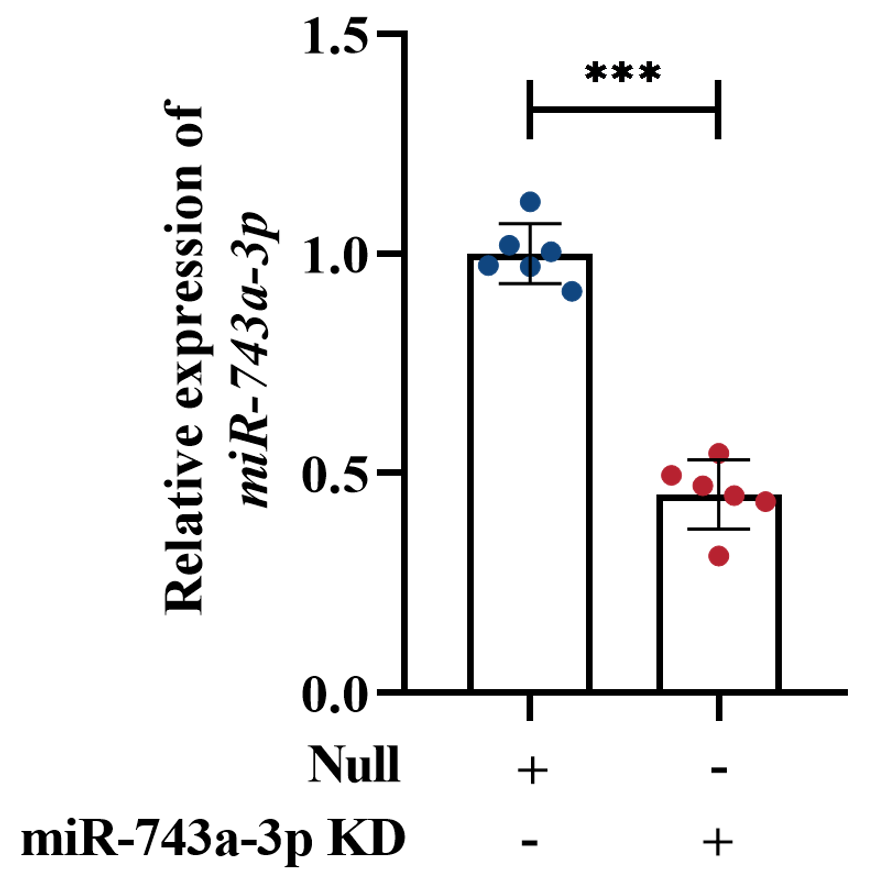


Fig. S5. The relative expression of hepatic miR-743a-3p was assayed in liver-specific miR-743a-3p knockdown mice. Data are presented as means ± SD. **p* <0.05, ***p* <0.01, ****p* <0.001 vs. corresponding control. (n = 6).


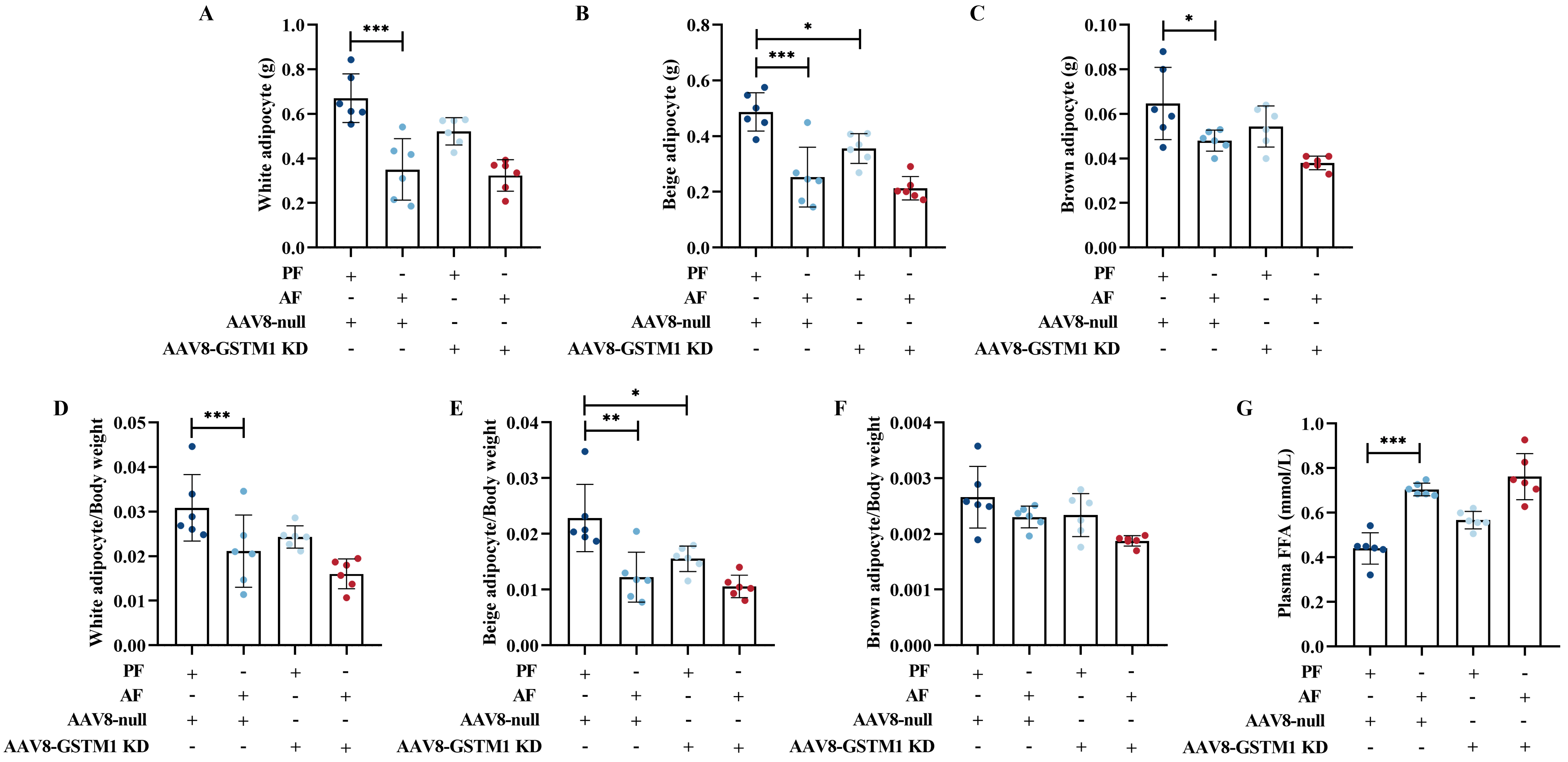
**Fig. S6**

Fig. S6. Liver-specific GSTM1 knockdown did not enhance alcohol-stimulated lipolysis in adipose tissue. (A) White adipocyte weight. (B) Beige adipocyte weight. (C) Brown adipocyte weight. (D) White adipocyte weight to body weight ratio. (E) Beige adipocyte weight to body weight ratio. (F) Brown adipocyte weight to body weight ratio. (G) Plasma FFA. Data are presented as means ± SD. **p* <0.05, ***p* <0.01, ****p* <0.001 vs. corresponding control. PF, pair-fed; AF, alcohol-fed. A-G (n = 6).

**Additional tables**

Table S1. Primers design for Real-Time PCR (mouse).

| Gene name | | Forward (5’-3’) | Reverse (5’-3’) |
| --- | --- | --- | --- |
| *18S* | AGGTCTGTGATGCCCTTAGA | | GAATGGGGTTCAACGGGTTA |
| *GSTM1* | GATCACCCAGAGCAACGCCATC | | AATGTCCACACGAATCTTCTCCTCTTC |
| *Acc* | GTTCTGTTGGACAACGCCTTCAC | | GGAGTCACAGAAGCAGCCCATT |
| *Srebp1c* | CCACCTCGTGAACGAAGATT | | ACCACCACGACCTCGAATAG |
| *Fasn* | GGAGGTGGTGATAGCCGGTAT | | TGGGTAATCCATAGAGCCCAG |
| *Scd1* | CGAGGGTTGGTTGTTGATCT | | GCCCATGTCTCTGGTGTTTT |
| *Cd36* | ATGGGCTGTGATCGGAACTG | | GTCTTCCCAATAAGCATGTCTCC |
| *Fatp2* | TCCTCCAAGATGTGCGGTACT | | TAGGTGAGCGTCTCGTCTCG |
| *Pparα* | TGCCTTCCCTGTGAACTGAC | | TGGGGAGAGAGGACAGATGG |
| *Ampk* | CAGGCCATAAAGTGGCAGTTA | | AAAAGTCTGTCGGAGTGCTGA |
| *Cpt1* | AAAAGTCTGTCGGAGTGCTGA | | GGCTTTCGACCCGAGAAGA |
| *Dgat2* | AGTGGCAATGCTATCATCATCGT | | TCTTCTGGACCCATCGGCCCCAGGA |
| *Vldlr* | GGAGATGCGATGGTGAAAAT | | AGAGGTGCTGCACTGGAACT |
| *Atgl* | CAACGCCACTCACATCTACGG | | GGACACCTCAATAATGTTGGCAC |
| *Hsl* | TCCCTCAGTATCTAGGCCAGA | | GGCTCATTTGGGAGACTTTGTTT |
| *Tnf-α* | CCCTCACACTCAGATCATCTTC | | GTTGGTTGTCTTTGAGATCCAT |
| *Il-1β* | GAAATGCCACCTTTTGACAGTG | | TGGATGCTCTCATCAGGACAG |
| *Il-6* | GATGCTACCAAACTGGATATAATC | | GGTCCTTAGCCACTCCTTCTGTG |
| *Mcp-1* | GGAAAAATGGATCCACACCTTGC | | TCTCTTCCTCCACCACCATGCAG |
| *Col1a1* | TGTGTTCCCTACTCAGCCGTCT | | CATCGGTCATGCTCTCTCCAA |
| *α-sma* | GTCCCAGACATCAGGGAGTAA | | TCGGATACTTCAGCGTCAGGA |
| *U6* | CTCGCTTCGGCAGCACATATAC | | AATATGGAACGCTTCACGAATTTG |
| *miR-324-3p* | AACGATACCACTGCCCCAGG | |  |
| *miR-505-5p* | ACTGGGAGCCAGGAAGTATTGATGTT | |  |
| *miR-706* | ACATTGGCAGAGAAACCCTGTC | |  |
| *miR-192-5p* | CGAGCTGACCTATGAATTGACAGCC | |  |
| *miR-743a-3p* | ACACTACGGAAAGACACCAAGC | |  |
| *miR-7088-3p* | AGTTGACCTTCCTCCATTGCTTCC | |  |
| *miR-1943-5p* | AACCACTAAGGGAGGATCTGGG | |  |
| *miR-1195* | AATATGTGAGTTCGAGGCCAGC | |  |
| *miR-1954* | CCAGCGACTGCAGAGTGAGA | |  |

Table S2. Antibodies list.

| Name | Citation | Supplier | Cat no. |
| --- | --- | --- | --- |
| GSTM1 | WB/IP | Santa Cruz Biotechnology | sc-517262 |
| P-ASK1 | WB | Affinity | AF8096 |
| ASK1 | WB | Affinity | AF6477 |
| P-JNK | WB | Cell Signaling Technology | 9255S |
| JNK | WB | Cell Signaling Technology | 3708S |
| P-p38 | WB | Santa Cruz Biotechnology | sc-7973 |
| p38 | WB | Huabio | ET1702-65 |
| β-actin | WB | Abclonal | AC026 |
| β-tubulin | WB | BOSTER | BM1453 |
| Goat anti-Mouse IgG | WB | BOSTER | BA1050 |
| Goat anti-Rabbit IgG | WB | BOSTER | BA1054 |
| F4/80 | IF | Cell Signaling Technology | 30325 |
| Alexa Fluor 488-labeled Goat anti-Rabbit IgG | IF | Beyotime | A0423 |

Table S3. Kits list.

| Name | Supplier | Cat no. |
| --- | --- | --- |
| H&E staining kit | G-Clone | RS3390 |
| Oil red O staining kit | Solarbio | G1261 |
| Sirius red staining kit | G-Clone | RS1220 |
| ALT assay kit | Nanjing Jiancheng | C010-2-1 |
| AST assay kit | Nanjing Jiancheng | C009-2-1 |
| TG assay kit | Abcam | Ab65336 |
| MDA assay kit | Beyotime | S0131 |
| GST assay kit | Solarbio | BC0355 |
| Dual luciferase assay kit | GenePharma | G06001 |
| Electrogenerated chemiluminescence kit | Vazyme | E422 |
| Reverse transcription reagent kit | Monad | MR05101 |

**Additional References**

1. Ding Q, Cao F, Lai S, et al. Lactobacillus plantarum ZY08 relieves chronic alcohol-induced hepatic steatosis and liver injury in mice via restoring intestinal flora homeostasis. *Food Research International*. 2022;157:111259.

2. Klaunig JE, Goldblatt PJ, Hinton DE, Lipsky MM, Chacko J, Trump BF. Mouse liver cell culture. *In Vitro*. 1981;17(10):913-925.

3. Hao L, Zhong W, Dong H, et al. ATF4 activation promotes hepatic mitochondrial dysfunction by repressing NRF1–TFAM signalling in alcoholic steatohepatitis. *Gut*. 2020;70(10):1933-1945.

4. Donohue TM, Osna NA, Clemens DL. Recombinant HepG2 cells that express alcohol dehydrogenase and cytochrome P450 2E1 as a model of ethanol-elicited cytotoxicity. *The International Journal of Biochemistry & Cell Biology*. 2006;38(1):92-101.

5. Sun J, Chen Y, Wang T. et al. Cadmium promotes nonalcoholic fatty liver disease by inhibiting intercellular mitochondrial transfer. *Cellular & Molecular Biology Letters*. 2023;28(1):87.
